# NF-κB modifies the mammalian circadian clock through interaction with the core clock protein BMAL1

**DOI:** 10.1371/journal.pgen.1009933

**Published:** 2021-11-22

**Authors:** Yang Shen, Mehari Endale, Wei Wang, Andrew R. Morris, Lauren J. Francey, Rachel L. Harold, David W. Hammers, Zhiguang Huo, Carrie L. Partch, John B. Hogenesch, Zhao-Hui Wu, Andrew C. Liu

**Affiliations:** 1 Department of Physiology and Functional Genomics, University of Florida College of Medicine, Gainesville, Florida, United States of America; 2 Department of Radiation Oncology and Center for Cancer Research, University of Tennessee Health Science Center, Memphis, Tennessee, United States of America; 3 Division of Human Genetics and Immunobiology, Cincinnati Children’s Hospital Medical Center, Cincinnati, Ohio, United States of America; 4 Department of Chemistry and Biochemistry, University of California Santa Cruz, Santa Cruz, California, United States of America; 5 Department of Pharmacology and Therapeutics, University of Florida College of Medicine, Gainesville, Florida, United States of America; 6 Department of Biostatistics, College of Public Health & Health Professions, University of Florida College of Medicine, Gainesville, Florida, United States of America; Charité - Universitätsmedizin Berlin, GERMANY

## Abstract

In mammals, the circadian clock coordinates cell physiological processes including inflammation. Recent studies suggested a crosstalk between these two pathways. However, the mechanism of how inflammation affects the clock is not well understood. Here, we investigated the role of the proinflammatory transcription factor NF-κB in regulating clock function. Using a combination of genetic and pharmacological approaches, we show that perturbation of the canonical NF-κB subunit *RELA* in the human U2OS cellular model altered core clock gene expression. While RELA activation shortened period length and dampened amplitude, its inhibition lengthened period length and caused amplitude phenotypes. NF-κB perturbation also altered circadian rhythms in the master suprachiasmatic nucleus (SCN) clock and locomotor activity behavior under different light/dark conditions. We show that RELA, like the clock repressor CRY1, repressed the transcriptional activity of BMAL1/CLOCK at the circadian E-box cis-element. Biochemical and biophysical analysis showed that RELA binds to the transactivation domain of BMAL1. These data support a model in which NF-kB competes with CRY1 and coactivator CBP/p300 for BMAL1 binding to affect circadian transcription. This is further supported by chromatin immunoprecipitation analysis showing that binding of RELA, BMAL1 and CLOCK converges on the E-boxes of clock genes. Taken together, these data support a significant role for NF-κB in directly regulating the circadian clock and highlight mutual regulation between the circadian and inflammatory pathways.

## Author summary

The circadian clock coordinates daily cell physiology. There has been considerable interest in identifying mechanisms that link cell physiology to circadian time-keeping. NF-κB is a major network hub controlling innate immunity and inflammation. Chronic constitutive activation of NF-κB is one of the primary causes of a number of human diseases and conditions such as immune diseases, metabolic disorders, neurodegenerative diseases, cancer and aging. Here we investigated the role of NF-κB in regulating the central and peripheral circadian clocks. Using a combination of genetic and pharmacological approaches we show that NF-κB perturbation alters clock oscillations in cells and tissues, as well as in mice. Further, using biochemical and biophysical methods, we show that NF-kB directly binds to the transactivation domain of BMAL1 where clock coregulators CBP/p300 and CRY bind; and as a result, like CRY, NF-kB represses E-box transcription. Together, these results support a significant role for NF-kB in linking inflammation to circadian timekeeping.

## Introduction

Endogenous circadian clocks allow organisms to coordinate behavior, physiology and metabolism to align with the external light-dark cycle [1]. Circadian clocks are present in virtually all cells of the body and are coordinated by the master clock in the suprachiasmatic nucleus (SCN) of the hypothalamus [2,3]. The SCN receives photic inputs and relays the light/dark information to peripheral tissues via neural and endocrine signals. In this manner, the SCN coordinates the peripheral oscillators into a coherent timing system [3]. The circadian clock regulates physiological functions in various tissues in mammals, including the immune and inflammatory responses [4–6]. Given the physiological importance of circadian timing, it is not surprising that its disruption is associated with a variety of pathological conditions and disease states, including neurodegenerative disorders, metabolic disorders, cardiovascular diseases, cancer, and aging [7–9].

In individual cells, the molecular clock is based on a transcriptional/translational negative feedback mechanism, in which activators drive the expression of their own repressors [10]. More specifically in mammals, the bHLH-PAS domain-containing transcriptional activators BMAL1 and CLOCK form a heterodimeric complex, which binds to the circadian E-box cis-elements to activate transcription of target genes, including Period (*PER1*, *2*, *3*) and Cryptochrome (*CRY1*, *2*). The PER/CRY proteins form their own complexes, and upon translocation to the nucleus, suppress BMAL1/CLOCK activity. This core loop interacts with at least two other feedback loops mediated by the circadian D-box and RORE cis-elements. These loops serve to stabilize the core loop and increase system robustness. The molecular clock regulates thousands of output genes that govern cell physiology, largely in a tissue-specific manner [10,11].

While the core clock mechanism is well understood and significant progress has been made in characterizing clock outputs, there exist additional clock components and modifiers that provide inputs to regulate the clock function. In an earlier effort to identify additional clock factors, we carried out a genome-wide RNAi screen in a human U2OS cell model and identified hundreds of genes whose knockdown impacted clock function [12]. These genes represent many cellular processes and signaling pathways that serve as inputs and link cellular functions to the clock [12,13]. These input pathways reflect the extensive interplay between the clock and cell physiology. Several emerging examples include nutrient/energy levels and redox stress [13,14]. In this study, we focused on how the pro-inflammatory nuclear factor-kappa B (NF-κB) pathway impacts clock function. NF-κB transcription factors play critical roles in immunity, inflammation, cell proliferation, differentiation, and survival [15,16]. NF-κB consists of a family of five related transcription factors: RELA (p65), RELB, c-Rel, NFKB1 (p105/p50), and NFKB2 (p100/p52). RELA and RELB each contain a transactivation domain and form dimers with p50 and p52, respectively, with the RELA/p50 complex representing the canonical pathway.

Proinflammatory stimuli such as tumor necrosis factor alpha (TNFα), interleukin 1 (IL-1) and bacterial lipopolysaccharides (LPS) induce the canonical NF-κB pathway. Upon stimulation, the cytosolic RELA/p50 complex translocates to the nucleus to mount a rapid response through transcriptional induction of target genes [15–17]. As a gatekeeper, inhibitor of NF-κB (IκBα, encoded by *NFKBIA*) serves as a repressor of NF-κB by virtue of masking its nuclear localization signal and preventing its nuclear entry. IκBα is regulated by the IκB kinase complex (IKK1, IKK2, and NEMO) that phosphorylates and targets IκBα for proteasomal degradation, thereby freeing RELA/p50 to enter the nucleus. In the non-canonical RELB/p52 pathway, p100 is phosphorylated by IKK1 and then converted to p52; the RELB/p52 dimer subsequently translocates to the nucleus [15,16]. In this manner, RELA and RELB use different mechanisms to regulate distinct physiological functions.

Here, we characterized the effect of the canonical RELA pathway on the circadian clock. Using genetic and pharmacological approaches, we show that RELA activation altered the expression patterns of core clock genes, resulting in altered circadian rhythms in cellular models and the SCN clock, as well as circadian locomotor behavior. RELA repressed E-box-mediated BMAL1/CLOCK transcriptional activity at a steady-state level. Biochemical and biophysical assays revealed a direct interaction between the REL homology domain (RHD) of RELA and the C-terminal regulatory domain (CRD) of BMAL1, centering on the transactivation domain (TAD). Our data suggest that NF-κB competes with CRY1 and coactivator CBP/p300 for binding to the BMAL1 TAD. Together, these results support a significant role for NF-κB in modulating circadian clock function. Given that inflammation and innate immunity are under the control of the circadian clock [5,18], our findings highlight the mutual regulation between these two pathways.

## Results

### NF-κB alters circadian rhythms in a cellular clock model

**Fig 1:**
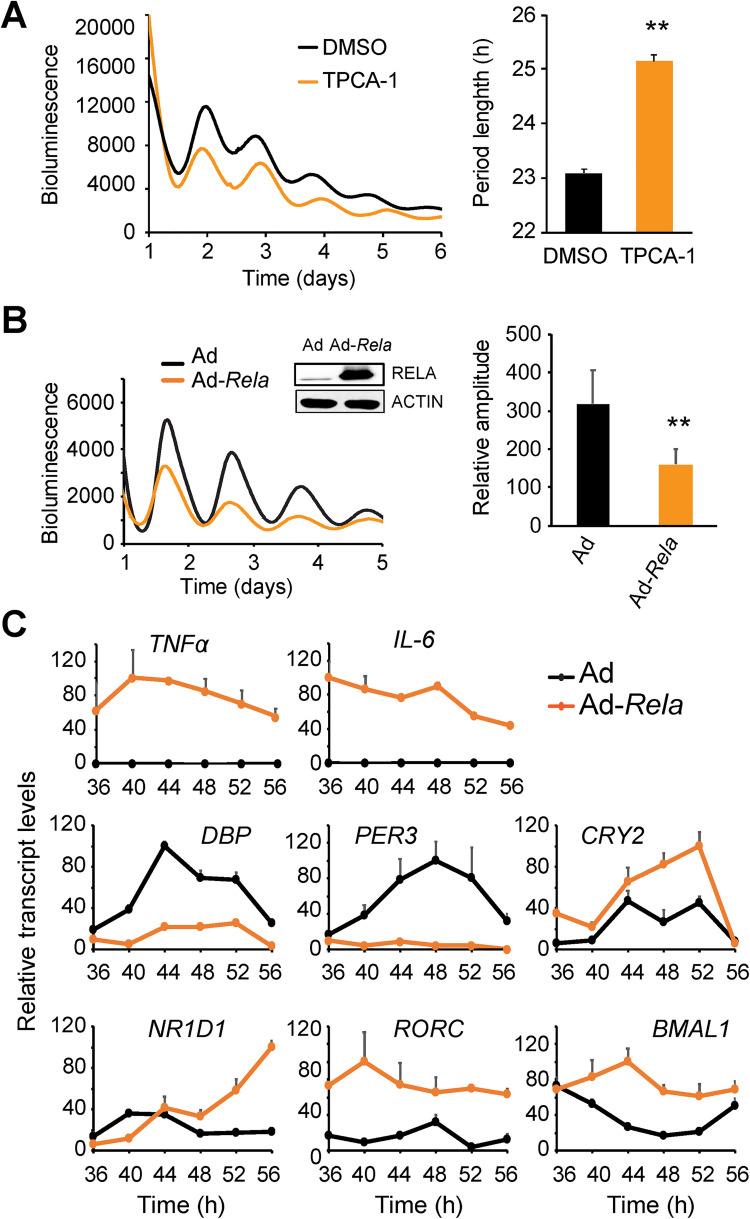
NF-κB affects clock gene expression and circadian rhythms in U2OS cells. **(A-B)** Bioluminescence rhythms in U2OS cells harboring the *Per2-dLuc* reporter were recorded in a Lumicycle luminometer. In (A), cells were treated with IKK2-specific inhibitor TPCA-1 (5 μM). IKK2 inhibition caused long period length compared to DMSO control. Traces are representative bioluminescence recordings. Period lengths are mean ± standard deviation (SD) of n = 6 independent samples for each condition. ** p<0.01. In (B), cells were transduced with an adenoviral *Rela* expression vector. Ad, adenoviral vector control; Ad-*Rela*, RELA adenoviral expression vector. Traces are representative bioluminescence recordings. Western blots (insert) were from protein extracts prepared one day after synchronization. Rhythm amplitudes in the bar graph are mean ± SD of n = 6 independent samples for each condition. ** p<0.01. **(C)** RELA overexpression alters clock gene expression in U2OS cells as in (B). (C) Ad or Ad-*Rela* transduced U2OS cells were synchronized by dexamethasone (Circadian time or CT0), and after 36 h, cell samples were harvested at 4 h intervals between CT36-CT56. Transcript levels for NF-κB target genes (*Tnfα* and *Il-6*) and core clock genes (*Dbp*, *Per3*, *Cry2*, *Nr1d1*, *Rorc* and *Bmal1*) were determined by Q-PCR and normalized to *GAPDH*. Error bars represent mean ± SD of 3 independent samples.

In an earlier functional genomic screen, we identified the NF-κB pathway as a clock modifier in U2OS cells expressing a *Per2-dLuc* reporter, in which a rapidly degradable *Luciferase* gene (*dLuc*) is under the control of the mouse *Per2* promoter [12]. For example, knockdown of *IκBα* and *IKK2* (to activate and inhibit the NF-κB pathway, respectively) in these cells caused short and long periods, respectively (S1A Fig). Consistent with the effect *of IκBα* knockdown and resultant NF-κB pathway activation, overexpression of a constitutively active *IKK2* mutant *IKK2*-S177/181E (or *IKK2*^*CA*^) to activate the pathway [19], resulted in an even shorter period length, compared to WT *IKK2* (S1B Fig). We then used several commonly used NF-κB pathway inhibitors to complement the genetic approach. TPCA-1 is a selective ATP-competitive inhibitor of IKK2 [20,21]. Withaferin A (WA) directly interacts with IKK2 to inhibit its catalytic activity, resulting in stabilized IκBα and reduced nuclear NF-kB and transcriptional activity [22,23]. CAT-1041, a conjugate of a polyunsaturated fatty acid and salicylic acid, inhibits NF-κB at least in part through IκBα stabilization [24]. Consistent with the *IKK2* knockdown effect, continuous exposure to TPCA-1 (Fig 1A), WA (S1C Fig), or CAT-1041 (S1D Fig) also caused longer periods in these cells, compared to DMSO control. Interestingly, while these inhibitors at high doses reduced the rhythm amplitude, CAT-1041 at lower doses increased the amplitude in these cells (S1D Fig).

To more directly assess the effect of NF-κB on the clock function, we tested whether overexpression of the canonical NF-κB subunit RELA affects circadian oscillations. For this, we used the adenoviral system to overexpress the mouse *Rela* gene in U2OS cells. Compared to cells transduced with the empty vector (Ad), cells expressing Ad-*Rela* displayed significantly decreased amplitude and a shorter period length (Fig 1B), without adversely impacting cell viability (S1E Fig). These results suggest that NF-κB affects clock function. Taken together, our genetic and pharmacological data support a role for NF-κB in regulating cell-autonomous circadian clocks.

### NF-κB alters core clock gene expression in U2OS cells

To determine how RELA affected circadian oscillations, we measured the expression patterns of core clock genes by quantitative PCR. We focused on the time window of 1.5–2.5 days (or 36–56 h) post synchronization when the clock phenotype was apparent but still maintained rhythmicity. As expected, *IL-6* and *TNFα*, the two classic NF-κB targets, were significantly upregulated in Ad-*Rela* cells, compared to Ad control (Fig 1C), suggesting that the exogenous RELA is functional in these cells. While the core clock genes in control cells displayed normal circadian expression patterns particularly their distinct phases, these patterns were altered in Ad-*Rela* cells (Figs 1C and S1F). RELA drastically reduced *DBP* and *PER3* expression at all circadian times. This result is consistent with previous reports showing that LPS and TNFα, known to induce NF-κB activation, caused low levels of *DBP* in the liver and lung [25–27]. *DBP* and *PER3* are known to be regulated by the E-box [10,28–30]. Further, *PER3* is also regulated by the D-box and the blunted DBP (D-box activator) likely contributed to the low *PER3* expression. Surprisingly, *PER2* expression was not drastically altered (S1F Fig), which is consistent with the robust rhythm at the time of sample collection. This data also suggests that the endogenous *PER2* and the *Per2-dLuc* reporter are regulated differently. In contrast, *BMAL1* was dramatically elevated at all circadian times (Fig 1C**)**. *BMAL1* is known to be controlled by the RORE cis-element and its expression in Ad-*Rela* cells may be explained by high levels of the RORE activator *RORC* and low levels of the RORE repressor *NR1D1*: while *NR1D1* was down-regulated during peak hours (36–40 h), its expression was abnormally high during trough hours (52–56 h); in contrast, *RORC* and *RORA* levels were abnormally high throughout the circadian cycle (Figs 1C and S1F). These expression patterns are consistent with previously reported cytokine effects [25–27].

### NF-κB alters circadian rhythms in the SCN clock and locomotor behavior

**Fig 2:**
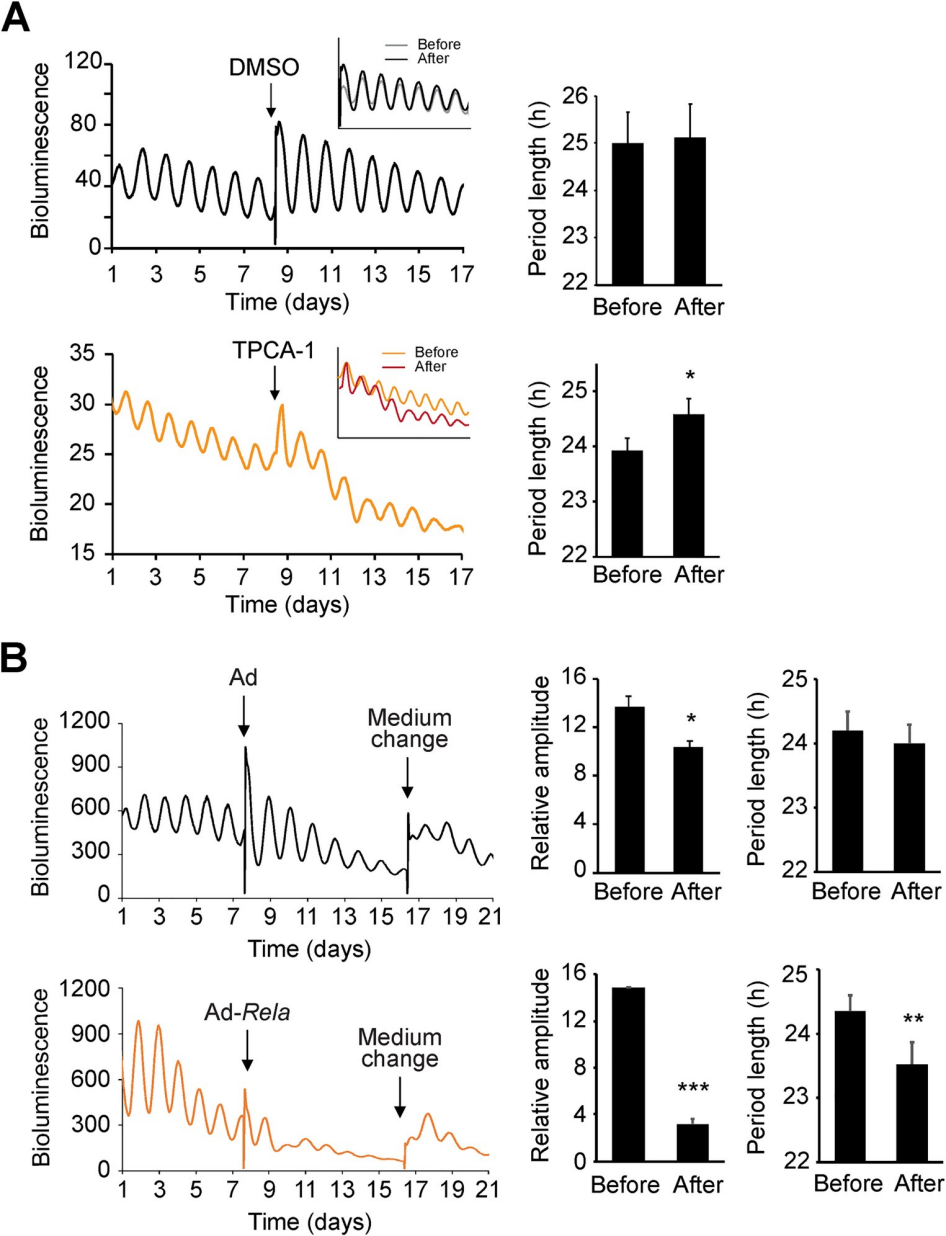
NF-κB affects circadian rhythms in the SCN. **(A)** Circadian bioluminescence rhythms of SCN explants from *Per2*^*Luc*^ reporter mice treated with IKK2 specific inhibitor TPCA-1 (5 μM) or DMSO as control. Period lengths are mean ± standard deviation (SD): 23.93 h ± 0.22 before treatment; 24.58 h ± 0.29 after treatment (n = 4 SCN slices of 4 different mice). The ‘before’ and ‘after’ treatment traces were plotted together for direct comparison (insert). **(B)** Circadian bioluminescence rhythms of *Per2*^*Luc*^ SCN explants expressing RELA. Ad, adenoviral vector control; Ad-*Rela*, adenoviral RELA expression vector. Compared to vector control, RELA overexpression reduced rhythm amplitude and shortened period length. Amplitude and period length data are mean ± SD (n = 5 SCN slices dissected from 5 different mice). * p<0.05, ** p<0.01, *** p<0.001. Data from additional SCN explants are included in S2 Fig.

The NF-κB effect on cell-autonomous clocks raised the possibility that it also affects the master SCN clock. Indeed, we show that treatment with TPCA-1 in SCN explants derived from PER2::LUC fusion (*Per2*^*Luc*^) knockin mice [31] lengthened rhythm period length compared to DMSO control (Fig 2A). To further test this, we infected SCN slices cultured *ex vivo* with Ad-*Rela*. Compared to controls that showed slightly reduced amplitude, SCN slices transduced with Ad-*Rela* displayed significantly lower amplitude and some displayed rapid loss of rhythmicity (Figs 2B and S2). Transient infection did not adversely impact neuronal viability as overall bioluminescence from the firefly luciferase reporter maintained during recording and medium change restarted bioluminescence oscillations. The period length was significantly shorter during the first few cycles of transient rhythms (Fig 2B).

**Fig 3:**
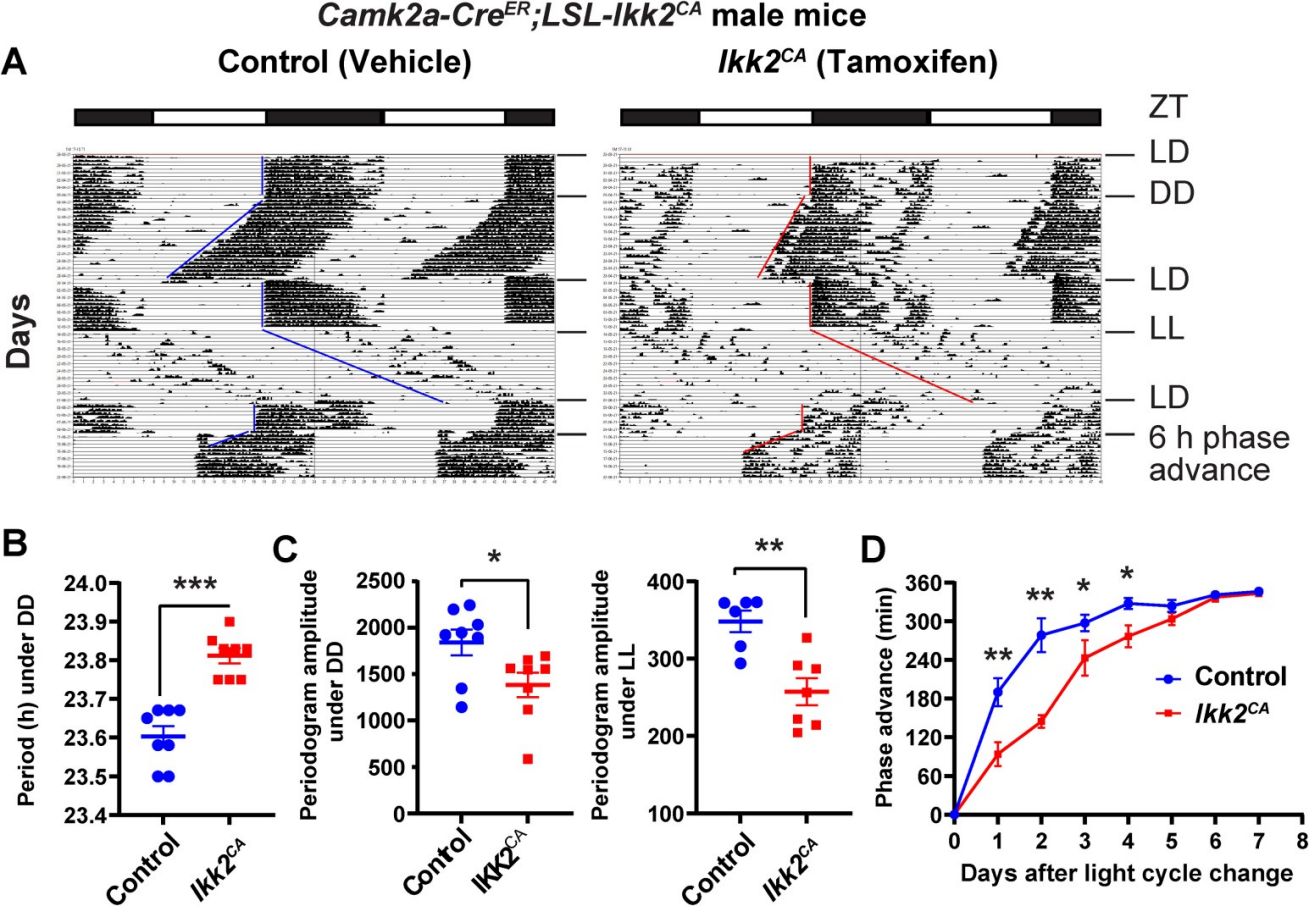
NF-κB affects circadian locomotor activity behavior. **(A)** Representative double-plotted actograms of wheel-running activity in vehicle control (corn oil) and *Ikk2*^*CA*^ male mice. X axis: zeitgeber time (ZT) of the 12 h/12 h light/dark cycle (LD) indicated by the bar (top). Y axis: number of days during the experiment. Mice were first entrained to a regular LD cycle and then released to constant darkness (DD) or constant light (LL). For phase shifting, mice were first entrained to LD and then the LD cycle was advanced for 6 h. **(B)** Circadian free-running period length in DD. **(C)** Periodogram amplitude of wheel-running behavior in mice under DD (left) and LL (right). **(D)** Line graphs showing the daily phase advance of wheel-running activity following a 6 h advancing LD cycle shift. Values are mean ± standard error (SE) (n = 7–8 mice for each group). * p<0.05. ** p<0.01. *** p<0.001 *Ikk2*^*CA*^ mice vs. vehicle by ANOVA.

Circadian animal behavior is an overt output of the SCN clock [3,32,33]. The NF-κB effect on the SCN clock raises the possibility that NF-κB affects the circadian locomotor activity. To test this, we obtained the *Camk2a-Cre*^*ER*^*;LSL-Ikk2*^*CA*^ mouse line. In the *LSL-Ikk2*^*CA*^ line, the *LoxP-Stop-LoxP (LSL)* cassette prevents expression of the constitutively active Flag-tagged *Ikk2-S177E/S181E (Ikk2*^*CA*^*)* and *IRES*-mediated *GFP* [34]. In the *Camk2a-Cre*^*ER*^ *line*, Cre^ER^ is conditionally expressed in neural tissues including forebrain and the hypothalamus [35,36]. Tamoxifen treatment induced deletion of the floxed LSL stop cassette and consequently conditional expression of *IKK2*^*CA*^ and GFP activation [34], which was confirmed by Western blot, FACS and immunofluorescence analyses (S3A–S3C Fig). We performed the wheel-running locomotor activity assay to assess circadian behavioral phenotypes. Corn oil vehicle control and tamoxifen-induced *Ikk2*^*CA*^ mice were entrained to the standard 12h/12h light/dark (LD) cycle for 2 weeks, followed by release to constant darkness (DD) to monitor their endogenous locomotor activity. Like the control mice, *Ikk2*^*CA*^ mice were able to entrain to the LD cycle and displayed robust free-running rhythms of locomotor activity in DD (Figs 3A and S3D). Under the DD condition, the circadian period in *Ikk2*^*CA*^ sex matched mice was longer than in the controls (Fig 3B; control: 23.60 h ± 0.03, n = 8; *Ikk2*^*CA*^: 23.81 h ± 0.02, n = 8; p < 0.0001), indicative of an altered SCN clock function. We also observed sex-dependent differences, in which the period length changes in males were more prominent than in females (S3E Fig).

After DD, mice were re-entrained to LD followed by release to LL. Mice under DD had more robust periodogram amplitudes than in LL, with control mice exhibiting higer amplitudes than mutant mice (Figs 3A and 3C and S3F, left panels), demonstrating stronger rhythmicity in the controls in LL. While weakly rhythmic under LL, control mice had a lengthened period length (25.15 h ± 0.22, n = 8). However, *Ikk2*^*CA*^ mice were largely arrhythmic under LL, with significantly lower periodogram amplitude relative to controls (Figs 3A and 3C and S3F, right panels).

To further characterize the circadian behavior of *Ikk2*^*CA*^ mice, we used an experimental “jet lag” regimen to study the phase shift response. For this, mice were kept in LD, followed by an abrupt 6 h phase advance. All mice were gradually re-entrained to the new LD cycle after 6 days. Notably, *Ikk2*^*CA*^ mice re-entrained more slowly than the controls. Following LD phase advance, *Ikk2*^*CA*^ mice exhibited a smaller phase shift than the controls (Fig 3A and 3D). These data suggest that NF-kB activation also altered light resetting and re-entrainment behavior.

### NF-κB represses BMAL1/CLOCK transcriptional activity

**Fig 4:**
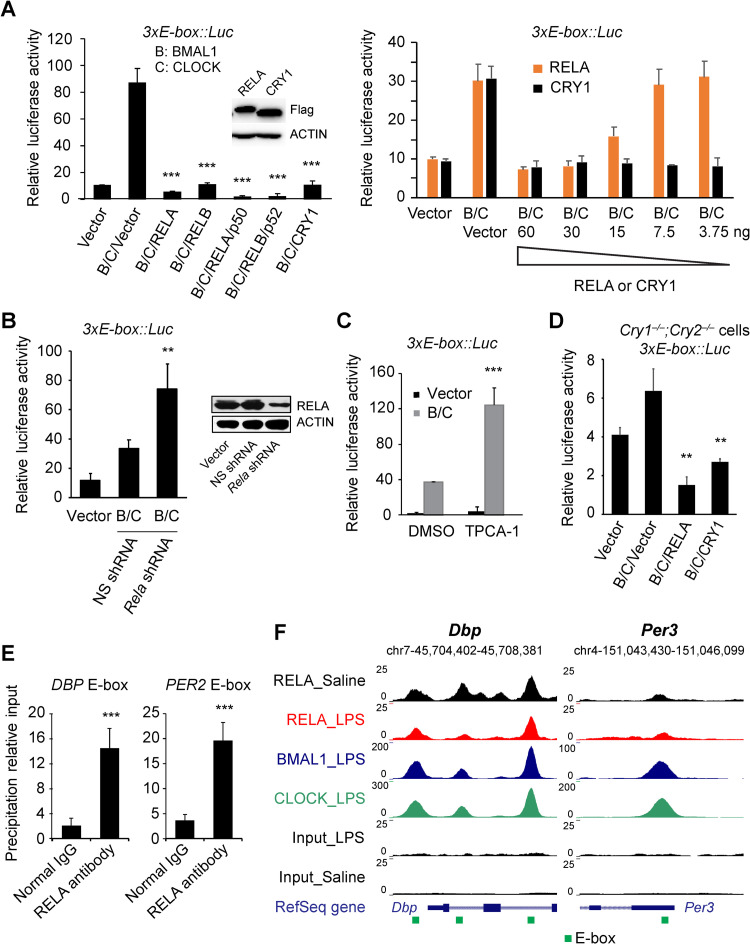
NF-κB represses E-box mediated transcriptional activity of BMAL1/CLOCK. **(A)** Steady-state luciferase reporter assay in transiently transfected 293T cells. In the *3xE-box*::*Luc* reporters, Luc expression is controlled by tandem E-box repeats. RELA and RELB repressed the transcriptional activity of BMAL1/CLOCK at the *3xE-box*::*Luc* reporter (left). While both RELA and CRY1 dose dependently repressed the reporter, CRY1 repression is more potent. Flag-tagged RELA and CRY1 were expressed to similar levels (Western blot inset). **(B-C)** RNAi knockdown of endogenous *RELA* (Western blot inset in B) or inhibition of IKK2 by TPCA-1 (20 μM) (C) relieved the repression of BMAL1/CLOCK by RELA. **(D)** Steady-state luciferase reporter assay in transiently transfected *Cry1*^*–/–*^*;Cry2*^*–/–*^fibroblasts using the *3xE-box*::*Luc* reporter. RELA was able to repress E-box transcription in the absence of the endogenous CRY. **(E)** RELA associates with the E-box cis-element of clock genes *PER2* and *DBP* in U2OS cells. Chromatin immunoprecipitation (ChIP) and Q-PCR to detect binding of endogenous RELA to the promoters of *PER2* and *DBP* at the E-box. ChIP was performed with an anti-RELA or normal IgG antibody. Representative data from three independent experiments are shown. Data are mean ± standard deviation (n = 3). **(F)** Representative UCSC genome browser images of RELA, BMAL1, and CLOCK ChIP-seq tracks at the *Dbp* and *Per3* genes. Normalized tag counts are indicated on the Y-axis. Green square, E-box. ChIP-seq analysis revealed overlapping binding of BMAL1, CLOCK and RELA at the E-boxes of *Dbp* and *Per3*. Motif analysis did not find NF-κB-RE in these binding peaks, suggesting indirect binding of NF-κB to the E-box. ** p<0.01. *** p<0.001.

The dramatic reduction of E-box genes such as DBP in cells overexpressing RELA raises the possibility that *NF-κB* more directly affects E-box transcription. To determine the RELA effect on E-box transcription, we performed steady-state reporter assays in transiently transfected HEK 293T cells. We used the *Per2*::*Luc* and *3xE-box*::*Luc* reporters, in which Luciferase (*Luc*) expression is driven either by the mouse *Per2* promoter that contains the E-boxes or by three tandem E-box repeats, respectively [37–39]. As expected, BMAL1 and CLOCK activated E-box transcription and CRY1 effectively repressed it in both reporters (Figs 4A and S4A). RELA and RELB, known as transcriptional activators, did not activate E-box transcription (S4A *Fig*). Both RELA and RELB effectively repressed BMAL1/CLOCK activity (Fig 4A, left panel), without adversely impacting cell viability (S4B Fig). RELB was previously shown to repress E-box expression [40] and our results show that RELA displayed higher E-box repression than RELB. Further, while both RELA and CRY1 exhibited dose-dependent repression of the *3xE-box*::*Luc* reporter, CRY1 displayed a more potent repression activity than RELA (Fig 4A, right panel). In this assay, RelA and Cry1 are under the control of the same promoter and were expressed to similar levels, suggesting that their repression activity was not due to differential expression (Fig 4A, western blot inset).

There is a considerable level of endogenous NF-κB in 293T cells. We reason that this basal activity would repress E-box transcription. In support of this notion, we show that shRNA knockdown of endogenous *RELA* relieved this repression, leading to significantly higher levels of reporter activity (Fig 4B). Similar to RNAi knockdown, inhibition of endogenous IKK2 with TPCA-1 also relieved its repression of BMAL1/CLOCK activity (Fig 4C). Other inhibitors of the NF-κB pathway such as GSK143, Bay11-7082 and CAT-1041 had similar effects (S4C Fig). This is consistent with the observation that low-dose NF-kB inhibitor CAT-1041 increased the rhythm amplitude (S1D Fig).

CRY1 and CRY2 are the canonical repressors of BMAL1/CLOCK. It is possible that NF-κB’s E-box repression is dependent on CRY1 or CRY2. To test this, we performed the reporter assay in *Cry1*^*–/–*^*;Cry2*^*–/–*^mouse fibroblasts [37]. In these *Cry*-deficient cells, the endogenous BMAL1 and CLOCK activated the *3xE-box*::*Luc* reporter, which was further upregulated by cotransfected BMAL1 and CLOCK and suppressed by RELA (Fig 4D). These results indicate that NF-κB represses E-box transcription in a CRY-independent manner.

### NF-κB regulates clock genes at the BMAL1/CLOCK-E-box loci

Our data support a model that NF-κB is recruited to the E-boxes of clock genes to repress BMAL1/CLOCK transcription. To test this prediction, we performed chromatin immunoprecipitation (ChIP) and quantitative PCR to determine the action of the endogenous RELA on clock genes *PER2* and *DBP* that have validated E-boxes [30,41]. We show that, compared to IgG control, RELA displayed enriched binding to the E-boxes in the promoters of *DBP* and *PER2* (Fig 4E).

Our data are consistent with a recent study showing that NF-κB targeted *Per2* and *Dbp* in the mouse liver upon LPS treatment [25]. Given the rich ChIP-seq data in that study, we mined the publicly accessible datasets to further define how NF-κB coordinates with BMAL1/CLOCK to regulate the E-box genes. ChIP-seq analysis revealed different gene cohorts targeted exclusively by RELA or by BMAL1/CLOCK, but not both. For example, the canonical NF-κB targets such as *Nfkb1*, *Nfkb2*, *Nfkbia*, and *Rela*, displayed significant RELA binding (S5A and S5B Fig and S1 Table). Importantly, these genes were not targeted by BMAL1 or CLOCK and no consensus E-boxes were found in the regulatory regions. Similarly, other genes such as *Ccne1*, *Gmfb*, *Atxn3* and *Aven*, contain E-boxes but not NF-κB-RE, and these sites were bound by BMAL1 and CLOCK, not by RELA.

Among the peaks bound by BMAL1 (6,114), CLOCK (22,247) and RELA (13,740), 1647 showed binding by all three factors, representing triple overlapping binding. All the core clock genes that are known to be regulated by BMAL1 and CLOCK via the E-box are represented in this list, including *Per1*, *Per2*, *Per3*, *Cry1*, *Cry2*, *Dbp*, *Nr1d1/Rev-erbα*, *Nr1d2/Rev-erbβ*, *Rorc/Rorγ*, and *Ciart/Chrono* (Figs 4F and S5C and S5D and S1 Table), as well as *Tef*, *Nfil3/E4bp4*, *Bhlhe40/Dec1* and *Bhlhe41/Dec2* (S1 Table). Among these genes, *Per1* and *Per2* peaks harbor both E-box and NF-κB-RE (S5C Fig and S1 Table), suggesting that their transcription may be regulated by RELA either directly through binding to NF-κB-RE or indirectly through binding to the BMAL1/CLOCK complex. Importantly, however, the triple binding peaks in other clock genes contain E-boxes only, but no consensus NF-κB-REs, indicative of indirect RELA binding on these chromatin sites. These data strongly support our model that NF-κB indirectly represses E-box transcription through complex formation with BMAL1/CLOCK at the E-box.

### NF-κB interacts with BMAL1 in the BMAL1/CLOCK complex

Given that many genes (e.g. *TNFα*, *IL-6*, *BMAL1*) are upregulated by RELA (Fig 1C), NF-κB’s repression on the E-box is unlikely caused by general transcriptional repression. Although known as an activator in the inflammatory response, RELA can repress transcription of some target genes in an HDAC-dependent manner [42]. However, inhibition of HDACs with chemical inhibitors such as nicotinamide and valproic acid did not attenuate RELA repression of E-box transcription (S4D Fig), arguing against an HDAC-dependent mechanism. Previous studies detected interactions between RELA and CLOCK at NF-κB target genes [43], and between RELB and the BMAL1/CLOCK complex [40]. Our data also show that RELA associated with the E-boxes of clock genes (Fig 4E). *As the 3xE-box*::*Luc reporter does not contain* NF-κB cis-response elements and alone is not responsive to NF-κB, it is likely that NF-κB affects E-box transcription via an indirect mechanism. In light of these observations, we hypothesize that RELA repression occurs through direct interactions with BMAL1 and/or CLOCK.

**Fig 5:**
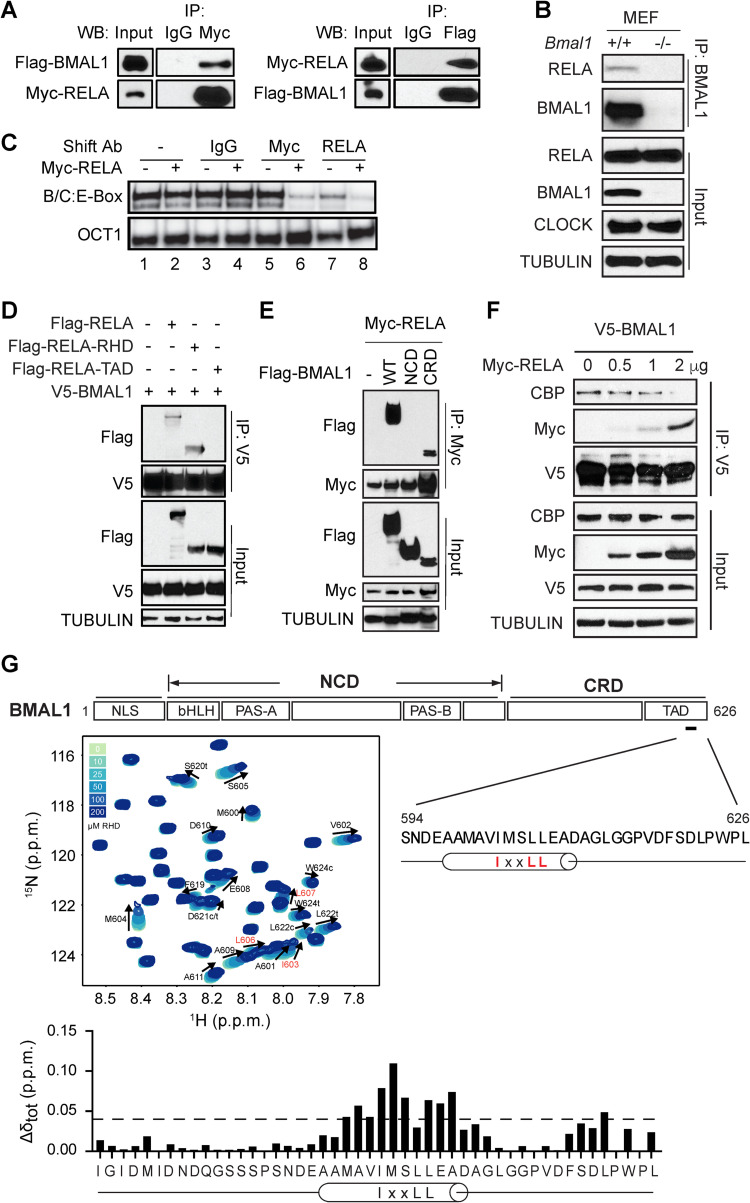
The RELA RHD interacts with the C-terminal transactivation domain of BMAL1. **(A-B)** Co-immunoprecipitation assays detect the interaction between RELA and BMAL1 in co-transfected 293T cells (A) and between the endogenous proteins in fibroblasts (B). The intrinsic interaction between RELA and BMAL1 is independent on CLOCK. **(C)** RELA associates with the BMAL1/CLOCK:E-box complex, indicative of a larger complex formation which caused a supershift. Oct1 oligonucleotide was used as control. The complex was reduced by the anti-Myc antibody, likely caused by a supershift that was not revealed in the gel. **(D-E)** Co-immunoprecipitation and Western blot analysis to detect the interaction between the RELA RHD and the BMAL1 CRD. 293T cells were co-transfected with tagged BMAL1 and RELA proteins as indicated. **(F)** Co-immunoprecipitation and Western blot analysis to show competition for interaction with BMAL1 between CBP and RELA. **(G)** Backbone chemical shift perturbations (Δδ_tot_) of ^15^N-BMAL1 C-terminal TAD with stoichiometric RELA RHD. Top panel: Schematic diagram of domain structure of BMAL1. The N-terminal core domain (NCD) contains the bHLH, PAS-A and PAS-B domains. The C-terminal regulatory domain (CRD) contains a transactivation domain (TAD) near the C-terminus featuring the highly conserved IxxLL helical motif. Residues within the IxxLL helical motif showed chemical shifts, indicative of interaction with RELA. Middle: Central region of ^15^N-^1^H HSQC spectra showing titration of 100 μM ^15^N-BMAL1 TAD in the presence of increasing concentrations of the RELA RHD (light to dark blue; solid arrow). Peak assignments are indicated for residues in the BMAL1 TAD that undergo chemical shift perturbation upon the addition of RELA RHD. Bottom: Quantification of backbone chemical shift perturbations (Δδ_TOT_) of ^15^N-BMAL1 TAD from the titration point with 200 μM RELA RHD. Dashed line, Δδ_TOT_ significance cutoff of 0.04 p.p.m. (parts per million).

We show that Myc-RELA interacts with Flag-BMAL1 by co-immunoprecipitation (co-IP) and Western Blot analysis (Fig 5A). The RELA and CLOCK interaction was weak but appeared stronger in the presence of BMAL1 (S6A–S6C Fig). Co-IP detected an endogenous interaction between BMAL1 and RELA in WT and *Clock*^*–/–*^cells, but not in *Bmal1*^*–/–*^cells (Figs 5B and S6D). These results suggest that the complex formation is dependent on BMAL1, but not CLOCK, and the RELA and CLOCK interaction occurred indirectly through BMAL1.

Using electrophoretic gel mobility shift assay (EMSA), we show that both endogenous and ectopically expressed BMAL1 and CLOCK formed a complex with a radio-labeled 14 bp E-box duplex probe (Figs 5C and S6E). As shown, this ternary complex at the E-box required both BMAL1 and CLOCK, as the complex was absent in mouse fibroblasts deficient in *Bmal1* or *Clock* (S6E Fig). This binding was effectively competed out with 100-fold excess of unlabeled (or cold) wild type E-box oligomer, but not with cold mutated E-box, confirming the specificity of the E-box complex formation (S6E Fig). The complex in the EMSA did not show a noticeable difference with or without Myc-RELA, suggesting that RELA itself does not disrupt the BMAL1/CLOCK:E-box complex (Fig 5C, lanes 1–2). An anti-Myc antibody, but not normal IgG, significantly decreased the complex signal, suggesting that Myc-RELA is part of the BMAL1/CLOCK:E-box complex (Fig 5C, compare lane 4 with lane 6). The complex was reduced by the anti-Myc antibody, likely caused by a supershift that was not revealed in the gel. Indeed, anti-RELA antibody caused similar changes for both endogenous and exogenous RELA (Fig 5C, compare lanes 4 and 8, and lanes 3 and 7). Taken together, these data support a model in which NF-κB binds directly to the BMAL1/CLOCK:E-box complex to repress E-box transcription rather than inducing disassembly of the complex.

### The RELA RHD domain interacts with the BMAL1 C-terminal regulatory domain

NF-κB functions primarily as a transcriptional activator [42]. We asked whether the NF-κB effect on the E-box is dependent on its transcriptional activity. NF-κB contains an N-terminal Rel homology domain (RHD) and C-terminal transactivation domain (TAD) [15,16] (S7A Fig). Mutation of Serine 281 within the RHD to glutamate (S281E) abolishes NF-κB transcriptional activity [44]. Using the steady-state reporter assay, we show that RELA-S281E lost its transcriptional activity to induce the *NF-κB-RE*::*dLuc* reporter, but was still able to repress the *3xE-box*::*Luc* reporter, despite weaker repression than WT RELA (S7B and S7C Fig). To determine which RELA domain underlies its repression, we generated two truncation constructs, RELA-RHD and RELA-TAD. We found that RELA-RHD, but not RELA-TAD, retains E-box repression activity in the *3xE-box*::*Luc* reporter assay (S7C Fig). Taken together, these results suggest that the classic NF-κB transcriptional activator activity is not required for its repression of the E-box.

Since RELA and BMAL1/CLOCK form a complex on the E-box, we next determined the specific domains that underlie the interaction. Co-IP detected an interaction between BMAL1 and the RELA RHD, but not the TAD (Fig 5D). Conversely, we asked which BMAL1 region associates with RELA. The N-terminal core domain (NCD) of BMAL1 contains the bHLH and PAS domains, responsible for dimerization and DNA binding [45]. Our recent studies found that part of the C-terminal regulatory domain (CRD) coordinates binding to CRY1 and coactivator CBP/p300, and their dynamic interactions plays a critical role in enabling circadian oscillations [46,47]. Intriguingly, we show that, like CRY1, RELA also interacted with the BMAL1 CRD (Fig 5E). These data suggest that RELA impacts clock function likely through competitive binding with CBP/p300. To test this hypothesis, we performed co-IP and Western blot analysis and showed that V5-BMAL1 interacts with both Myc-RELA and CBP. Importantly, Myc-RELA was able to effectively compete with CBP for BMAL1 binding, with increasing amounts of Myc-RELA correlating with decreased amounts of CBP (Fig 5F). These results further support the mechanism in which RELA competes with CBP for binding to the BMAL1 CRD.

### NF-κB binds to the BMAL1 C-terminal transactivation domain

In our previous study, we showed that CRY1 and CBP compete for binding to the transactivation domain (TAD) within the BMAL1 CRD and the shared binding sites center on the IxxLL helical motif within the α-helix of the TAD and on a second cluster of residues in the distal C terminus (Fig 5G) [46,47]. Our results showing that NF-κB binds to the BMAL1 TAD raised the possibility that the same IxxLL motif is involved. To determine the precise binding site of NF-κB on the BMAL1 TAD, we collected ^15^N HSQC NMR spectra of ^15^N-labeled TAD in the presence or absence of the RELA RHD and showed that titration of the RELA RHD led to dose-dependent chemical-shift perturbations at residues within the helical motif of the TAD and the distal C terminus, overlapping directly with regions that bind CRY1 and CBP (Fig 5G). These results confirm the direct interaction between BMAL1 and NF-κB and demonstrate that CBP, CRY1 and NF-κB binding all converge on overlapping binding sites on the BMAL1 TAD.

## Discussion

### NF-κB is a circadian clock modifier

Our genome-wide RNAi screen uncovered many genes, representing many cellular pathways and functions, that provide input to and modify the clock [12]. The past decade has seen several examples of the integration of circadian clocks directly with cell physiology: AMP-activated protein kinase (AMPK) [48], mTOR and autophagy in response to nutrient and growth signals [49–53], NAD^+^-dependent deacetylase sirtuin-1 (SIRT1) [54–57], oxidative stress inducible transcription factor NRF2 [58,59], and hypoxia inducible factor HIF1α [60–62]. In the present study, we identified NF-κB as a clock modifier and characterized the mechanism of how NF-κB regulates circadian clock function. NF-κB appears to bind to the regulatory sequences of core clock genes to affect their expression even under non-stress conditions. We show that it represses E-box transcription through direct binding to the BMAL1 TAD. In the U2OS cellular clock model, NF-κB activation and inhibition shortened and lengthened circadian rhythm period lengths, respectively, whereas hyperactivation of NF-κB correlated with low amplitude phenotypes (Figs 1 and 2 and S1).

NF-κB perturbation also altered the SCN clock and circadian behavioral rhythms, as seen in *Ikk2*^*CA*^ (Figs 2 and 3 and S3) and *Ikk2*^*–/–*^mice [25]. The behavioral period phenotype of *Ikk2*^*CA*^ mice is modest but significant as observed in other mutant mouse models such as *Clock*^*–/–*^, *Nr1d1*^*–/–*^, *Chrono*^*–/–*^, *Mtor*^*+/–*^and *Tsc2*^*+/–*^mice [49,50,63–65], as well as in *Ikk2*^*–/–*^mice [25]. Behavioral phenotypes are usually less dramatic than those in cells and this robustness is attributable to SCN neural networking and intercellular coupling [3,32]. Findings from a previous study also suggested a role of NF-κB in the regulation of circadian rhythms [25]. The long period phenotype in *Ikk2*^*CA*^ mice (NF-κB constitutive activation) is mechanistically consistent with the short period phenotype observed in *Ikk2*^*–/–*^mice [25]. Taken together, basal NF-κB activity under normal conditions is important for maintenance of circadian homeostasis and increased NF-κB activity impacts the clock function. However, these behavioral phenotypes (e.g. long period in *Ikk2*^*CA*^ mice) are intriguing, as cells and SCN explants showed opposite period phenotype. This phenotypic difference between animal behavior and cell and tissue clocks (as in U2OS and SCN) suggest possible involvement of systemic signals such as those in the neuroendocrine and autonomic neural systems. Of relevance, there exists a reciprocal regulation between innate immunity and sleep disturbance [66], and constitutive NF-κB activation or inactivation may alter neuroimmunology at the organismal level, contributing to cell/tissue non-autonomous behavioral phenotypes.

### Mechanisms of E-box repression by NF-κB

NF-κB could affect clock function by several mechanisms: binding to NF-κB-REs present in clock genes to directly upregulate gene expression, recruiting HDACs to modify chromatin and repress transcription [42], and interacting with BMAL1/CLOCK bound at the E-box to indirectly repress gene expression. While upregulation of canonical NF-κB targets via the NF-κB-RE correlated with increased RNA Pol II binding and increased H3K27 acetylation, NF-κB repression of BMAL1/CLOCK activity at the E-box was correlated with decreased Pol II binding and decreased H3K27 acetylation [25]. It was suggested in that study that LPS affected the clock repressors such as *Per*, *Cry*, *E4bp4* and *Nr1d1*, not the *Bmal1*, *Clock*, *Dbp* and *Ror* activators. Importantly, however, the high-resolution ChIP-seq data revealed that most of the BMAL1/CLOCK/RELA triple binding peaks, including not only the clock repressors but also the activators such as *Dbp*, *Tef* and *Rorc*, contain only E-boxes, suggesting indirect binding of NF-κB at the E-box (Figs 4 and S5 and S1 Table). This is evident in the *3xE-box*::*Luc* reporter assay, in which the promoter sequence, unlike the endogenous genes, does not contain NF-κB-REs or other confounding factors (Fig 4). Further, genes such as *Bmal1* and *Clock* that are not regulated via the E-box did not show RELA binding. Intriguingly, RELA displayed binding peaks at the E-boxes in saline controls, independent of LPS treatment. Some of these peaks were slightly shifted by LPS and even displayed decreased binding for all three factors (Figs 4 and S5) [25]. These observations suggest that LPS-induced NF-κB has some remodeling effect on the BMAL1/CLOCK-E-box complex. Future studies are needed to investigate how BMAL1, CLOCK and NF-κB coordinate to regulate E-box transcription.

Compared to NF-κB transcriptional activation, its repression mechanism is much less understood [15,67]. Of relevance, acetylation of RELA by CBP/p300 at specific sites can have both positive and negative effects on DNA binding and transcription, and so do phosphorylation and dephosphorylation. Interestingly, RELA can also recruit HDACs that modify chromatin to repress transcription [42]. However, HDAC inhibitors did not affect RELA repression of BMAL1/CLOCK **(**S4D Fig), suggesting that HDAC activity is not required for E-box repression. Our work showed that the BMAL1 CRD coordinates interactions with CRY1 and CBP/p300 which plays a key role in enabling circadian oscillations [46,47]. In this study, we show that, like CRY1, RELA also interacts with the BMAL1 CRD. Thus, RELA impacts clock function likely through competitive binding with CRY repressors. Our data suggest that CRY1 is more potent than NF-kB in repressing E-box transcription. Recent studies have made important progresses in our mechanistic understanding of CRY repression which involves dynamic interactions with the BMAL1 CRD and CLOCK PAS domains [46,47,68,69]. As repressors of BMAL1/CLOCK at the E-box, RELA and CRY may share some aspects of the repression mechanism which warrant future studies.

### NF-κB underlies mutual regulation between circadian clocks and inflammation

Both the innate and adaptive immunological functions have prominent circadian rhythms, including gene expression, synthesis and secretion of cytokines and chemokines, and immune cell functions [5,18]. Within innate immunity, recent studies have documented prominent circadian rhythms in monocytes and macrophages [5]. Toll-like receptors and cytokine and chemokine display circadian oscillations. Several lines of evidence support circadian regulation of NF-κB activity. First, key players in the NF-κB pathway (e.g., IκBα) are rhythmically expressed in several peripheral tissues including the liver and lung, exhibiting a circadian phase similar to *Per1* [11,41]. Second, CLOCK was shown to be recruited by RELA at the NF-κB-RE to influence NF-κB target genes [43], adding another layer of circadian regulation of NF-κB activity. NF-κB signaling was altered in *Clock* mutant mice and in *Cry*-deficient mice [43,70]. In a different twist, RELA activation was shown to be regulated through CRY repression of adenylyl cyclase and consequently inhibition of PKA-mediated RELA phosphorylation [71]. RORα, a key activator of the RORE loop, upregulates IκBα expression, thereby hindering NF-κB nuclear translocation and activity [72]. Third, the *NF-κB-RE*::*dLuc* reporter assay showed that NF-κB displays circadian activities in the liver [43]. These studies support mutual regulation between the circadian and the inflammatory pathways.

Several studies in recent years have demonstrated the effects of inflammation on clock function. Inflammation induced by endotoxin treatment with LPS, IL-1 or TNFα altered clock gene expression in the SCN [26,73], the lung [27,74], liver [25,26,73], and fibroblasts [26], as well as locomotor activity [25,75–78]. LPS-induced lung and liver inflammation caused a reprograming of core clock genes and outputs and led to a short period length phenotype [25,27], consistent with our data. In a mouse model of chronic obstructive pulmonary disease (COPD), cigarette smoke exposure combined with viral infection disrupts clock function and leads to enhanced inflammatory responses in the lung and reduced locomotor activity [79]. Further, endotoxin was shown to suppress clock gene expression in human peripheral blood leukocytes [80]. At the molecular level, both RELA and RELB can directly interact with BMAL1 and repress BMAL1/CLOCK activity, thus providing a mechanistic basis for the inflammatory effect on circadian clock function. Our data suggest that, under normal conditions of immune and inflammatory homeostasis, basal NF-κB activity contributes to circadian homeostasis. Under endotoxin-induced inflammation, hyperactivated NF-kB competes with CRY1 to a certain degree to interfere with E-box function. Importantly, chronic constitutive activation of NF-κB is considered one of the primary causes of a number of human diseases including immune diseases, metabolic disorders, neurodegenerative diseases, cancer, and aging. Under these pathological conditions, heightened NF-kB activity would compete with CRY1, thereby impacting circadian timekeeping and likely sleep/wake homeostasis [66,81].

## Experimental procedures

### Ethics statement

Mice were housed in the animal facility at the University of Florida. All animal experiments were conducted according to the National Institutes of Health Guide for the Care and Use of Laboratory Animals and approved by the Institutional Animal Care and Use Committee at the University of Florida (No. 202110057).

### Plasmids

The expression constructs pLV7-CAG-1xFlag-*Bmal1* (full length), 1xFlag-*Bmal1*-NCD containing the bHLH, PAS-A and PAS-B domains, 1xFlag-*Bmal1*-CRD containing the G and H domains, *Bmal1*-V5, and 3xFlag-*Clock* were cloned as described previously [46,50]. The plasmids encoding Flag-*Rela* (plasmid #20012), Flag-*Relb* (#20017), Flag-*p50*(#20018) and Flag-*p52* (#20019) were obtained from Addgene. *Rela*-S281E mutant was generated with primers 5’-tctgat cgcgagctcgaggagcccatggagttc-3’ and 5’-gaactccat gggctcctcgag ctcgcgatcaga-3’. The PCR product was inserted into p3xFlag-CMV-10 vector at HindIII and EcoRI sites. *Rela*-RHD was generated with primers 5’-agctaagcttatggacgatctgtttcc-3’ and 5’-agctgcggc cgcttacatgatactcttgaaggtctc-3’, *Rela*-TAD with 5’-agctaagcttgagacctt caagagtatcatg-3’ and 5’-agctgcggccgcttaggagctgatctgactcaa-3’. The amplified products were cloned into p3xFlag-CMV-10 vector at HindIII and NotI sites. pLV7-CAG-*Ikk2* WT and *Ikk2*-S177/181E were generated as previously described [19,50]. The pGL3-*Per2*::*Luc* and pGL3-*3xE-box*::*Luc* reporters used in 293T transient transfection reporter assay were described previously [37,46,82].

For adenoviral gene expression, pShuttle-CMV (Addgene #16403) and AdEasier-1 cells (#16399) were purchased from Addgene. Full length *Rela* gene was inserted into pShuttle-CMV with SalI and NotI to obtain pShuttle-CMV-*Rela*. pShuttle-CMV and pShuttle-CMV-*Rela* were linearized with PmeI, and transformed into competent AdEasier-1 bacterial cells for recombination. The recombinants are named Ad for vector control and Ad-*Rela* for *Rela*. Recombinant adenoviruses were prepared and used as described previously [83].

### Animal and wheel-running locomotor activity assay

Mouse SCN organotypic slices were dissected and cultured in explant medium as previously described [32,50]. Mice conditionally expressing constitutively active IKK2 (*Ikk2*^*CA*^) in neural tissues including the forebrain and SCN (Camk2a-Cre^ER^;LSL-*Ikk2*^*CA*^ mice, abbreviated as *Ikk2*^*CA*^ mice) were generated by crossing the LSL-*Ikk2*^*CA*^ mice (in which a LoxP-flanked STOP cassette prevents transcription of the transgenes, Flag-tagged *Ikk2*^*CA*^ and IRES-mediated EGFP) [34] with the Camk2a-CreER mice [35] (gift of Dr. Sylvain Dore, Department of Anesthesiology). Both lines were obtained from the Jackson Laboratory. Mice of ~3 months old were treated with either corn oil (control) or tamoxifen (75 mg per kilogram of body weight) via i.p. injection for 5 consecutive days. Mice were used for experiments 2 weeks after tamoxifen treatment. Western blot and FACS analyses were performed to confirm transgene expression. Mice were individually housed in cages equipped with running wheels and locomotor activities were recorded as previously described [32,50]. Briefly, mice were entrained to a standard 12hr/12hr light/dark cycle for 2 weeks days and then released to constant darkness (DD) for 2–3 weeks. Wheel-running activities were recorded and analyzed using the ClockLab program (Actimetrics).

### Immunofluorescence staining and flow cytometry

Mice were anaesthetized with isoflurane and euthanized. The brain was perfused with PBS and 4% paraformaldehyde and dehydrated in 10%, 15% and 30% sucrose in PBS. Following dehydration, brains were frozen in OCT (Thermo Scientific) on dry ice and stored at −80°C. Brains were cut using a cryostat and 30 μm sections were mounted on Superfrost Plus microscope slides (Fisher Scientific). Slides were stored at −80°C. For staining, slides were washed with PBS, permeabilized with PBS+0.2% Triton X-100 (PBST) and blocked with 2% normal donkey serum (Abcam ab7475) in PBST for 1 h at room temperature. Slides were incubated with primary antibodies in 2% normal donkey serum in PBST overnight at 4°C. Primary antibodies used include 1:500 anti-NeuN (ab177487) and 1:2000 anti-GFP (ab13970). Slides were then washed and incubated with Alexa Fluor-conjugated secondary antibodies at 1:500 in 2% normal donkey serum in PBST for 1 h at room temperature. Secondary antibodies include Alexa Fluor 594 donkey anti-rabbit IgG H&L (ab150076) and Alexa Fluor 488-goat anti-chicken IgY H&L (ab150173). Slides were washed and cover-slipped using Prolong Gold anti-fade with DAPI (Invitrogen) and dried overnight. Imaging was performed on a SP8 Confocal Microscope (Leica).

For brain cell dissociation and flow cytometry, mice were anesthetized and brain tissues dissociated with the Adult Brain Dissociation Kit (Miltenyi Biotec, cat. # 130-107-677). The CNS tissue of each mouse was transferred to a gentle MACS C Tube (Miltenyi Biotec) containing both enzyme mixes from the kit for enzymatic digestion. Mechanical enzymatic tissue dissociation was performed using program 37C_ABDK_01 of the gentle MACS Octo Dissociator with Heaters (Miltenyi Biotec). After dissociation, each CNS homogenate was applied to one 70 μm cell strainer (Corning), followed by debris removal and red blood cell lysis following the kit. Flow cytometry was performed on total brain cells and GFP expression was analyzed on live single cell gated populations.

### Cell culture, transfection and steady-state reporter assays

Cell culture and growth conditions for HEK 293T and U2OS cells were performed as previously described [12,46,84]. Briefly, 293T and U2OS cells were maintained in Dulbecco’s Modified Eagle Medium (DMEM) (HyClone) supplemented with 10% fetal bovine serum, as previously described [84,85]. For the reporter assay, 293T cells were cultured and seeded in 96-well plates, and transfected with desired plasmids using Lipofectamine 2000 (Invitrogen). Cells were harvested 24 h later and assayed with the Dual-Glo Luciferase Assay System (Promega). Firefly luciferase activity was normalized to Renilla luciferase as an internal control for transfection efficiency, as detailed in our previous studies [37,46,86]. The luminescent CellTiter-Glo ATP assay (Promega) was used to assess cell viability. For the Lumicycle assay, samples were collected at day 6 at the end of the run when the clock phenotype was at the strongest. For the transient transfection assay, samples were collected 24 h post transfection when the Dual-Glo Luciferase Assay was performed.

### Bioluminescence recording and data analysis

We used a LumiCycle luminometer (Actimetrics) for luminescence recording as previously described [39,50,59]. Briefly, cells were grown to confluence in 35-mm dishes and synchronized with 200 nM dexamethasone. After synchronization, the cells were washed with PBS and then replaced with luciferin-containing bioluminescence recording medium. For adenoviral infection, the cells were infected with adenovirus for 4 h and then changed to regular growth medium. One day later, the infected cells were synchronized with dexamethasome, washed thoroughly with PBS, and then replaced with recording medium, followed by the bioluminescence rhythm assay. The recording medium contained 1x DMEM, 10 mM HEPES, 144 mM NaHCO_3_, 1% fetal bovine serum, 1x B-27 and 0.5 mM luciferin, buffered to pH 7.4. Three independent dishes/samples were used for each condition. Raw luminescence data (counts/s) as a function of time (days) were analyzed with the LumiCycle Analysis program (version 2.53, Actimetrics) to determine circadian parameters. Normally, the first 12 h data are excluded in parameter analysis. Briefly, raw data were fitted to a linear baseline, and the baseline-subtracted data were fitted to a sine wave (damped), from which period length, goodness-of-fit, and damping constant were determined. For samples that showed persistent rhythms, goodness-of-fit of 90% was usually achieved. For amplitude analysis, raw data from last 3 days were fitted to a linear baseline, and the baseline-subtracted (polynomial number = 1) data were fitted to a sine wave, from which amplitude values were obtained.

### Quantitative PCR analysis

For quantitative PCR (qPCR) analysis, U2OS cells were synchronized using 200 nM dexamethasone for 2 h and infected with adenovirus expressing Ad vector control or Ad-*Rela*. 4 h later, cells were changed to regular DMEM culture medium. After 36 h, cells were harvested at 4 h intervals for a complete circadian cycle. RNA extraction, reverse transcription, and quantitative real-time PCR were performed as previously described [50,59,84]. SYBR Green PCR master mix (Thermo Scientific) was used in qPCR. The primers used in qPCR analysis are listed in S2 Table. Transcript levels for each gene were normalized to *GAPDH* and values were expressed as percentage of expression in control cells.

### Electrophoretic mobility shift assay

Electrophoretic mobility super-shift assay (EMSA) was performed as done previously [87]. In brief, 20 μg of total cell extract was incubated with ^32^P-labeled double-stranded oligonucleotides containing an E-box (CGCGCAAAGCCATGTGCTTCCCCCT) at room temperature for 20 min. The samples were resolved on acrylamide gel electrophoresis and quantified with a Cyclone phosphoimager (Perkin Elmer). For supershift assay, cell extracts were incubated with 1 μg of antibody in 10 μl of EMSA reaction buffer on ice for 30 min, followed by procedures for EMSA. Oct1 oligonucleotide (Promega) was used as control. Wild type and mutant E-box sequences used as cold competition probes are listed in S3 Table.

### Immunoprecipitation and immunoblotting

Cell lysate preparation and immunoblotting were performed as previously described [50,87–89] with minor modifications. Briefly, cells were harvested by trypsinization and immediately lysed in RIPA lysis buffer containing cocktails of proteases inhibitors (Roche) and phosphatase inhibitors (Sigma). The primary antibodies used in this experiment are as following: guinea pig antibodies against BMAL1 and CLOCK from Choogon Lee’s lab; rabbit antibodies against RELA (Cell Signaling), Myc (Cell Signaling), Flag (Sigma), HA (Sigma); and Actin and Tubulin were from Santa Cruz Biotech.

Immunoprecipitation was performed as previously described [90] with minor modifications. In brief, cells were lysed in 10% PBS and 90% IP lysis buffer (20 mM Tris, pH 7.0, 250 mM NaCl, 3 mM EDTA, 3 mM EGTA, 0.5% Nonidet P-40, 2 mM DTT, 0.5 mM PMSF, 20 mM β-glycerol phosphate, 1 mM sodium orthovanadate, 1 μg/ml leupeptin, 1 μg/ml aprotinin, 10 mM *p-*nitrophenyl phosphate, and 10 mM sodium fluoride). 5% of total lysate was used as input control samples. The rest of the lysate was incubated with 1 μg of primary antibody on a rotator at 4°C overnight. Next day, protein G-sepharose was added to the mixture and incubated at 4°C for 4 hr. Finally, protein G-sepharose enriched complexes were resolved on SDS-PAGE and analyzed by Western blot analysis.

Chromatin immunoprecipitation (ChIP) was performed as previously described [87,90] with minor modifications. U2OS cells were synchronized using 200 nM dexamethasone for 2 hours and then infected by adenovirus of Ad vector control or Ad-*Rela*. 4 hours later, cells were changed to regular DMEM culture medium. After 36 hours, cells were fixed for 10 min at room temperature in 1x PBS containing 0.5% formaldehyde and quenched with 0.125 M glycine for 15 min. After washing with cold PBS, the cells were scraped into 1x PBS in a 15 mL conical tube and pelleted at 1,500 rpm for 10 min at 4°C. The pellet was suspended and lysed in lysis buffer (5 mM PIPES, pH 8.0, 85 mM KCl, 0.5% Nonidet P-40) for 10 min on ice. Cell lysates were sonicated with eight 20-second pulses with 20-second pauses. Cell nuclei were pelleted at 1,300 rpm for 10 min at 4°C and resuspended in 1 mL nuclei lysis buffer (50 mM Tris-Cl, pH 8.0, 10 mM EDTA, 1% SDS, supplemented with protease inhibitors), followed by incubation on ice for 10 min and then sonication with six 15-second pulses with 45-second pauses. The samples were centrifuged at 15,000 rpm at 4°C for 5 min and supernatants were used for ChIP. Primers used in qPCR to amplify the E-box region of *PER2* and *DBP* genes were designed based on previous studies [30] and are listed in S4 Table.

### NMR spectroscopy

All proteins were expressed in *Escherichia coli* Rosetta2 (DE3) cells from a pET22b vector backbone. Recombinant proteins for RELA-RHD (1–318) and BMAL1 TAD (579–626) were cloned from the mouse *Rela* or *Bmal1* genes, and each had an N-terminal TEV-cleavable His-GST tag. Cells were grown in Luria Broth media (for natural abundance RELA-RHD) or M9 minimal media with ^15^N-ammonium chloride for uniform incorporation of the stable isotope for NMR of BMAL1 TAD. All cultures were grown at 37°C until an O.D._600_ of ~0.8 was reached, after which expression was induced with addition of 0.5mM IPTG. Cultures were grown at 18°C for an additional 16–20 hours. Cell pellets for the BMAL1 TAD were lysed in buffer containing 50 mM Tris, pH 7.5, 300 mM NaCl and 20 mM imidazole for affinity purification using Ni-NTA resin (Qiagen) as we have done previously [46]. Imidazole-eluted protein was buffer exchanged to a low-imidazole buffer by desalting column or diafiltration with an Amicon stirred-cell concentrator under nitrogen pressure. The His-GST tag was removed via TEV protease overnight at 4°C. Cleaved protein was separated from TEV and the His-GST tag with a Ni-NTA column and proteins were further purified by size-exclusion chromatography on Superdex 75 16/600 (GE Life Sciences) in NMR buffer (10 mM MES, pH 6.5, and 50 mM NaCl). For RELA-RHD, cells were lysed using a high-pressure extruder (Avestin) in buffer containing 50 mM Tris, pH 7.5, 300 mM NaCl, 1mM EDTA, 5 mM BME. Affinity purification was carried out with Glutathione Sepharose 4B resin (GE Healthcare). The His-GST tag was removed via TEV protease overnight at 4°C and cleaved protein was separated from the TEV and the His-GST tag with a Ni-NTA column. Protein was further purified with size-exclusion chromatography on HiLoad 16/600 Superdex 75 prep grade column (GE Healthcare) in NMR buffer. Proteins were aliquoted and frozen in liquid nitrogen for long-term storage at -80°C.

NMR experiments were conducted on a Varian INOVA 600-MHz spectrometer at 25°C equipped with ^1^H, ^13^C, ^15^N triple-resonance, z-axis pulsed-field-gradient probes. All NMR data were processed with NMRPipe/NMRDraw [91]. Chemical-shift assignments were obtained from previous work in the lab [46]. Concentrated RELA-RHD protein was added stepwise to 100 μM ^15^N-TAD protein for the ^15^N HSQC titration. Each sample was concentrated to 300 μL final volume and adjusted to a final concentration of 10% (v/v) D_2_O. ^15^N HSQC titration data were analyzed with CCPNMR [92] with chemical-shift perturbations defined by the equation Δδ_tot_ = [(Δδ^1^H)^2^ + (χ(Δδ^15^N)^2^]^½^ and normalized with the scaling factor χ = 0.15, established from estimates of atom-specific chemical-shift ranges in a protein environment [93].

### ChIP-sequencing analysis

We obtained previously reported ChIP-seq data with antibodies targeting on the RELA/p65 subunit of NF-κB, CLOCK, BMAL1, and their input sample (no specific antibody) of mouse liver treated with LPS from GEO (GSE117488) [25]. For comparison, we also obtained NF-κB antibody ChIP-seq data and its input sample from mice treated with saline. Each condition contained two biological replicates. The raw fastq reads were aligned to Mus musculus genome assembly (mm10) using Bowtie 2 with sensitive-local option [94], which can automatically trim the mismatched nucleotide bases (low quality or adaptors) from the end of a read. The biological replicates of the aligned reads were merged together before further analyses. FindPeaks function in the Homer software [95] was deployed to perform peak calling for the NF-κB, CLOCK, and BMAL1 samples with their respective input sample as background control. Significant peaks were declared using the default false discovery rate threshold 0.1%. The comparison of the peaks from NF-κB, CLOCK, and BMAL1 samples were conducted using the mergePeaks function in Homer. Selected peaks were visualized using UCSC genome browser [96]. The peak sequences of ~200 bp were used for transcription factor binding motif search using the JASPAR program with a relative profile score threshold of 85% or above [97].

## Supporting information

S1 Fig Perturbation of the NF-κB pathway components altered circadian rhythms in U2OS cells harboring the Per2-dLuc reporter. **(A)** siRNA-mediated knockdown of IκBα and IKK2 in U2OS cells caused short and long period lengths, respectively. **(B)** Exogenous expression of IKK2-S177/181E (IKK2^CA^) that leads to constitutive acitivation of NF-κB caused short period lengths in U2OS cells. **(C-D)** IKK2 inhibition with chemical inhibitors withaferin A (WA, 1 μM) in (C) or different doses of CAT-1041 in (D) caused long period lengths in U2OS cells. In (B-D), n = 3 independent wells. ** p < 0.01 relative to control. **(E)** ATP assay to assess cell viability. Cell samples were collected at day 5–6 at the end of the Lumicycle run when the clock phenotype was the strongest. Adenoviral RELA expression in U2OS cells did not cause toxicity or cell death. **(F)** NF-κB affected clock gene expression in U2OS cells. Cells were transduced with either adenoviral vector control (Ad) or NF-κB subunit RELA (Ad-*Rela*). The expression patterns of core clock genes were determined by Q-PCR. See Fig 1C for detail.
(TIF)

S2 Fig NF-κB affects circadian rhythms in the SCN. Circadian bioluminescence rhythms of *Per2*^*Luc*^ SCN explants expressing either adenoviral vector control (Ad) or adenoviral RELA expression vector (Ad-*Rela*). Shown are additional SCN explants for Fig 2B.
(TIF)

S3 Fig NF-κB affects circadian locomotor activity behavior in mice. **(A-B)** Characterization of *Camk2a-Cre*^*ER*^*;LSL-Ikk2*^*CA*^ mice. Mice treated with corn oil serve as vehicle controls. Tamoxifen injection leads to Cre-mediated deletion of the LSL stop cassette and allows expression of the Flag tagged *Ikk2*^*CA*^ transgene and the IRES-mediated GFP. Flag-IKK2^CA^ and IL12B (an NF-kB target) expression was detected by Western blotting (A) and GFP by FACS analysis (B). **(C)** Immunohistochemical staining to show neuronal expression of the transgene upon tamoxifen injection. Blue, DAPI; green, GFP; red, NeuN. SCN: suprachiasmatic neuclei. 3V, the third ventricle. Scale bars: 200 μm and 100 μm. **(D)** Representative double-plotted actograms of wheel-running activity rhythms in control and *Ikk2*^*CA*^ female mice. See Fig 3 for detail. **(E)** Circadian period length by sex. n = 4 mice for each group. *** p < 0.001. **(F)** Representative plots of periodogram amplitude of mice under constant darkness (DD, left panel) and constant light (LL, right panel). In control mice in LL, a main peak is seen at ~25 h, indicating lengthened period length. Compared to controls, *Ikk2*^*CA*^ mice more readily became arrhythmic and had lower periodogram amplitude.
(TIF)

S4 Fig NF-κB affects E-box-mediated transcription. Steady-state luciferase reporter assay in transiently transfected 293T cells. **(A)** NF-κB itself, in the absence of BMAL1/CLOCK (B/C), did not activate the E-box transcription from the *3xE-box*::*Luc* or *Per2-dLuc* reporters (left, middle), but repressed BMAL1/CLOCK activation of the *Per2*::*Luc* reporter (right). RELA, RELB, p50 and p52 are NF-κB subunits. **(B)** ATP assay to assess cell viability. Transfection of the NF-kB subunits in 293T cells did not cause toxicity or cell death. Cell samples were collected 24 h post transfection and used for the Dual-Glo Luciferase Assay. **(C)** Inhibition of the endogenous NF-κB by chemical inhibitors (10 μM GSK143, 20 nM Bay 11–7082, or CAT-1041 as the indicated doses) relieved its repression on the E-box-mediated transcription of the luciferase reporter. **(D)** HDAC inhibitors nicotinamide (1 mM) and valproic acid (10 mM) did not affect NF-κB repression of E-box transcription. n = 6 independent wells for each assay. ** p < 0.01.
(TIF)

S5 Fig Representative UCSC genome browser images of RELA, BMAL1, and CLOCK ChIP-seq tracks at the genes. ChIP-seq analysis revealed overlapping binding of BMAL1, CLOCK and RELA at the E-boxes of the indicated clock genes. **(A)** Examples of NF-kB targets via NF-kB-RE. **(B)** BMAL1/CLOCK targets via E-box. **(C)** Examples of core clock genes regulated by BMAL1/CLOCK via E-box and by NF-kB via NF-kB-RE. **(D)** Examples of core clock genes regulated by coordinated actions of BMAL1/CLOCK and RELA via E-box only. Normalized tag counts are indicated on the Y-axis. The genomic DNA for the region (chromosome # and sequence start and end position) is shown on the top. The predicted motif are shown on the bottom. Green square, E-box. Red square, NF-kB-RE. Motif prediction is in S1 Table. Note that although motif search for Nr1d1 predicted an NF-kB-RE site at the second peak, the sequence is located at the peak periphery, not at the peak center (S1 Table), and the peak was not LPS inducible, suggesting that NF-kB binding at the second peak is mediated by the two E-boxes at the peak center.
(TIF)

S6 Fig RELA associates with BMAL1 on the E-box. **(A)** Reciprocal co-IP and Western blot to detect the interaction between RELA and CLOCK. Their interaction was not consistently detected in cotransfected 293T cells even after long exposure, indicative of weak interactions. **(B)** Interaction between RELA and CLOCK was readily detected in co-transfected 293T cells in the presence of coexpressed BMAL1. **(C)** The RELA and CLOCK interaction was drastically reduced in Bmal1-deficient mouse fibroblasts. **(D)** Co-IP and Western blot detected BMAL1 and RELA interaction in Clock-deficient mouse fibroblasts. **(E)** Electrophoretic mobility shift assay (EMSA). The BMAL1/CLOCK (B/C) heterodimer formed a complex with ^32^P-labeled E-box duplex probe (B/C:E-box) in WT cells (left), but not in cells deficient in Bmal1 or Clock, indicating that the BMAL1/CLOCK dimer is required for the BMAL1/CLOCK:E-box ternary complex formation. The specificity of complex formation was confirmed by completion with a 100-fold excess of unlabeled (cold) wild type probe (WT1, 2), but not with mutated duplex (mutant).
(TIF)

S7 Fig RELA interacts with the BMAL1 C-terminal transactivation domain. **(A)** Schematic diagram of domain structure of RELA. RHD: Rel homology domain. TAD: transactivation domain. Shown are the point mutation and truncation constructs used in the study. **(B-C)** Steady state luciferase reporter assay in transiently transfected 293T cells using the *NF-κB-RE*::*dLuc* (B) or *3xE-box*::*Luc* reporter (C). The RELA-S281E mutant failed to activate the *NF-κB-RE*::*dLuc* reporter, but effectively repressed the *3xE-box*::*Luc* reporter. The RELA RHD retains the E-box repression activity. n = 6 independent wells. *** p < 0.001.
(TIF)

S1 Table The E-box and NF-kB-RE motifs within the binding peaks of BMAL1, CLOCK and RELA in ChIP-seq data. (PDF)

S2 Table Q-PCR primers for human genes. (PDF)

S3 Table Q-PCR primers for ChIP-PCR. (PDF)

S4 Table Probe sequences for gel shift. (PDF)
