# Supplementary figures and images for: NF-κB modifies the mammalian circadian clock through interaction with the core clock protein BMAL1
Source: PLoS Genet. 2021 Nov 22;17(11):e1009933. doi: 10.1371/journal.pgen.1009933 (PMC8648109; doi:10.1371/journal.pgen.1009933)

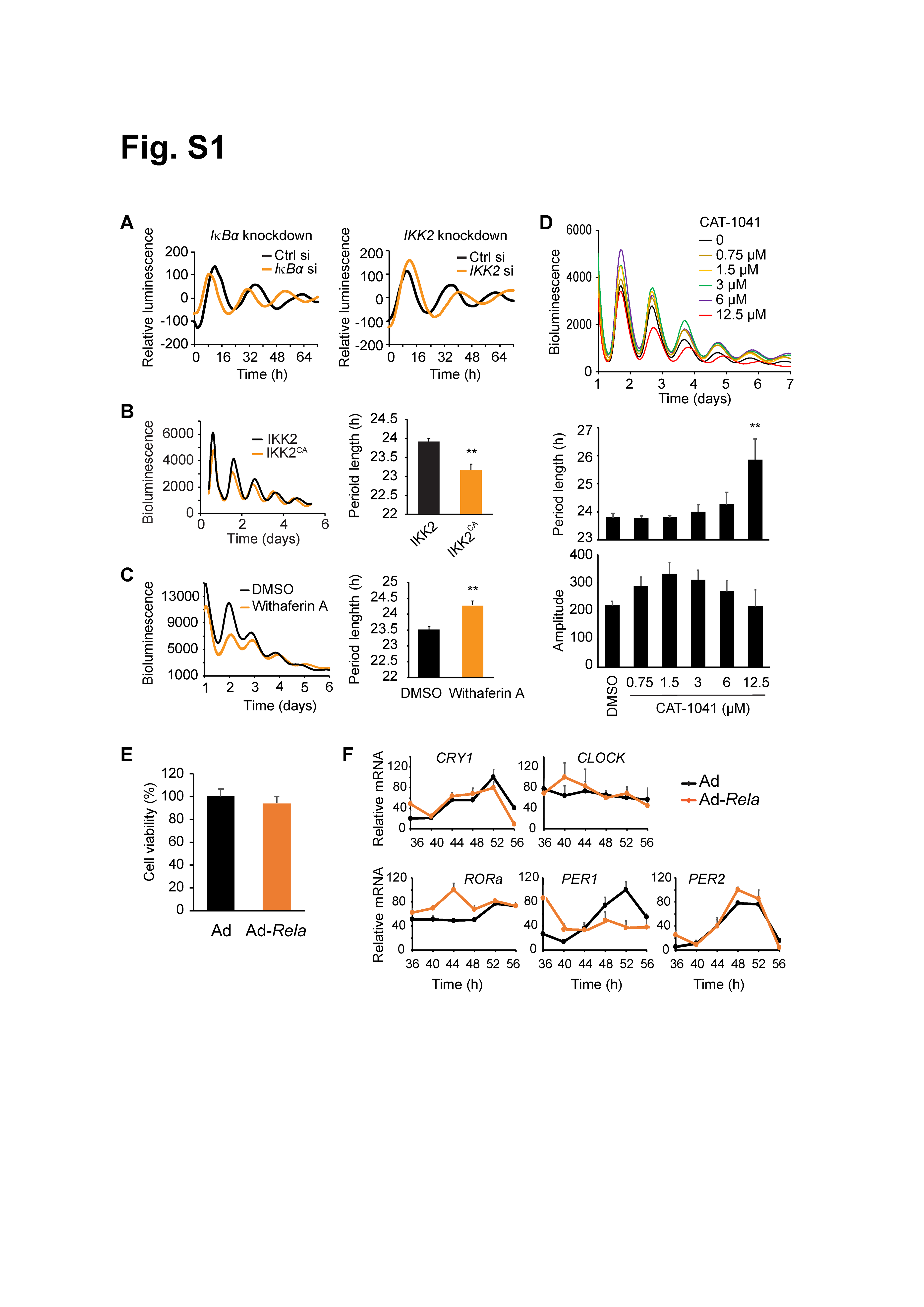

Supplement: S1 Fig — (A) siRNA-mediated knockdown of IκBα and IKK2 in U2OS cells caused short and long period lengths, respectively. (B) Exogenous expression of IKK2-S177/181E (IKK2CA) that leads to constitutive acitivation of NF-κB caused short period lengths in U2OS cells. (C-D) IKK2 inhibition with chemical inhibitors withaferin A (WA, 1 μM) in (C) or different doses of CAT-1041 in (D) caused long period lengths in U2OS cells. In (B-D), n = 3 independent wells. ** p < 0.01 relative to control. (E) ATP assay to assess cell viability. Cell samples were collected at day 5–6 at the end of the Lumicycle run when the clock phenotype was the strongest. Adenoviral RELA expression in U2OS cells did not cause toxicity or cell death. (F) NF-κB affected clock gene expression in U2OS cells. Cells were transduced with either adenoviral vector control (Ad) or NF-κB subunit RELA (Ad-Rela). The expression patterns of core clock genes were determined by Q-PCR. See Fig 1C for detail. (TIF) [file pgen.1009933.s001.tif]

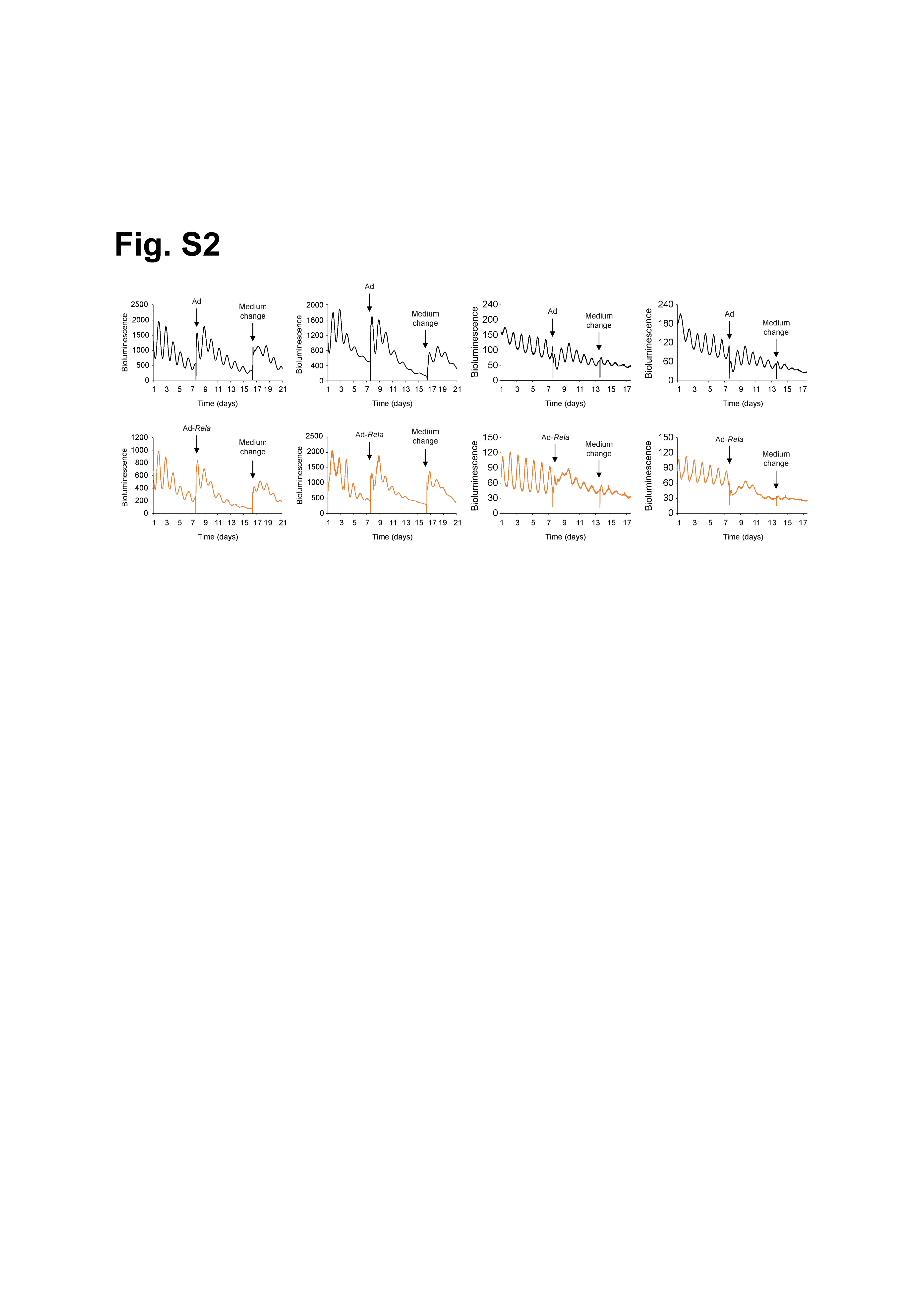

Supplement: S2 Fig — Circadian bioluminescence rhythms of Per2Luc SCN explants expressing either adenoviral vector control (Ad) or adenoviral RELA expression vector (Ad-Rela). Shown are additional SCN explants for Fig 2B. (TIF) [file pgen.1009933.s002.tif]

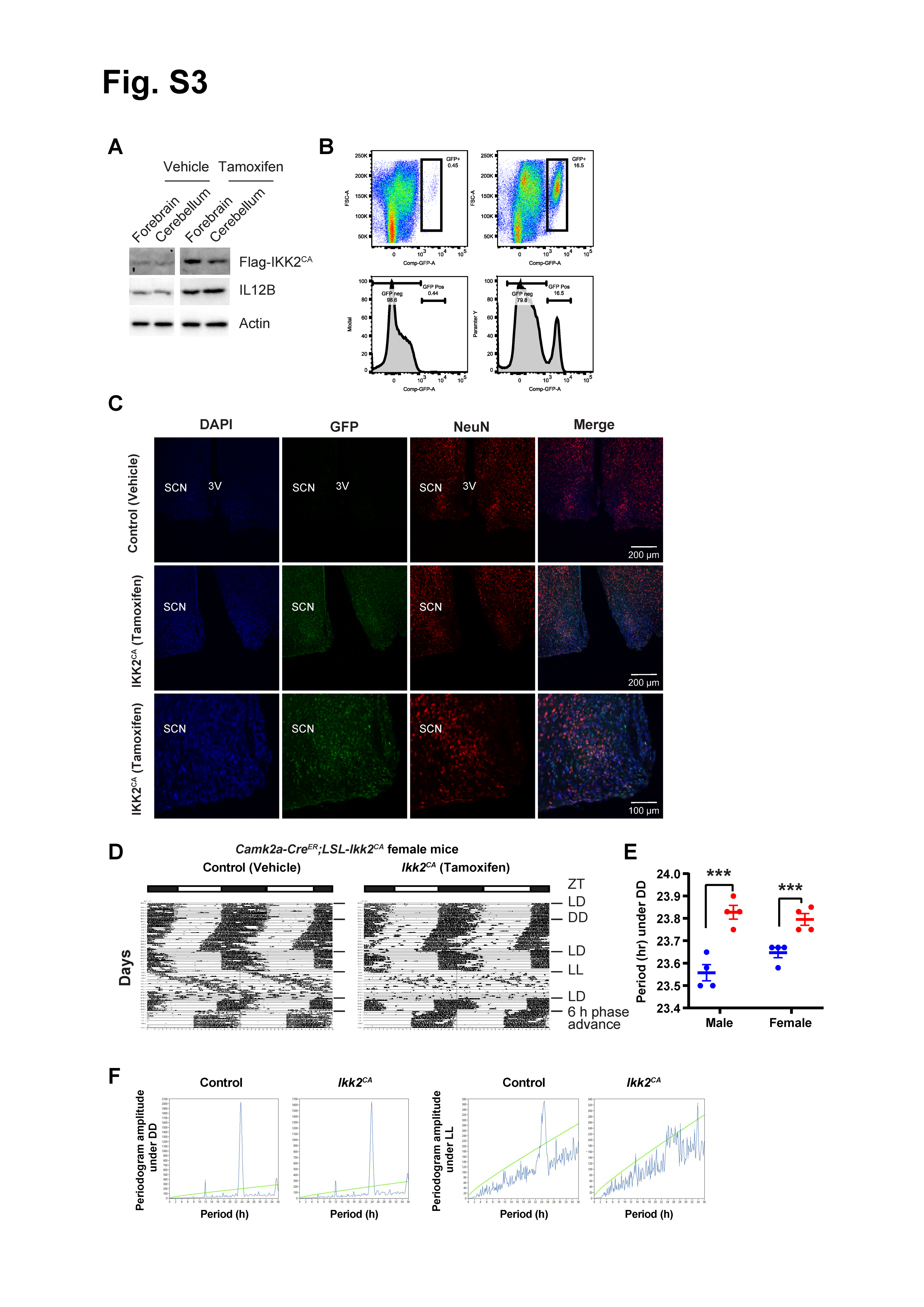

Supplement: S3 Fig — (A-B) Characterization of Camk2a-CreER;LSL-Ikk2CA mice. Mice treated with corn oil serve as vehicle controls. Tamoxifen injection leads to Cre-mediated deletion of the LSL stop cassette and allows expression of the Flag tagged Ikk2CA transgene and the IRES-mediated GFP. Flag-IKK2CA and IL12B (an NF-kB target) expression was detected by Western blotting (A) and GFP by FACS analysis (B). (C) Immunohistochemical staining to show neuronal expression of the transgene upon tamoxifen injection. Blue, DAPI; green, GFP; red, NeuN. SCN: suprachiasmatic neuclei. 3V, the third ventricle. Scale bars: 200 μm and 100 μm. (D) Representative double-plotted actograms of wheel-running activity rhythms in control and Ikk2CA female mice. See Fig 3 for detail. (E) Circadian period length by sex. n = 4 mice for each group. *** p < 0.001. (F) Representative plots of periodogram amplitude of mice under constant darkness (DD, left panel) and constant light (LL, right panel). In control mice in LL, a main peak is seen at ~25 h, indicating lengthened period length. Compared to controls, Ikk2CA mice more readily became arrhythmic and had lower periodogram amplitude. (TIF) [file pgen.1009933.s003.tif]

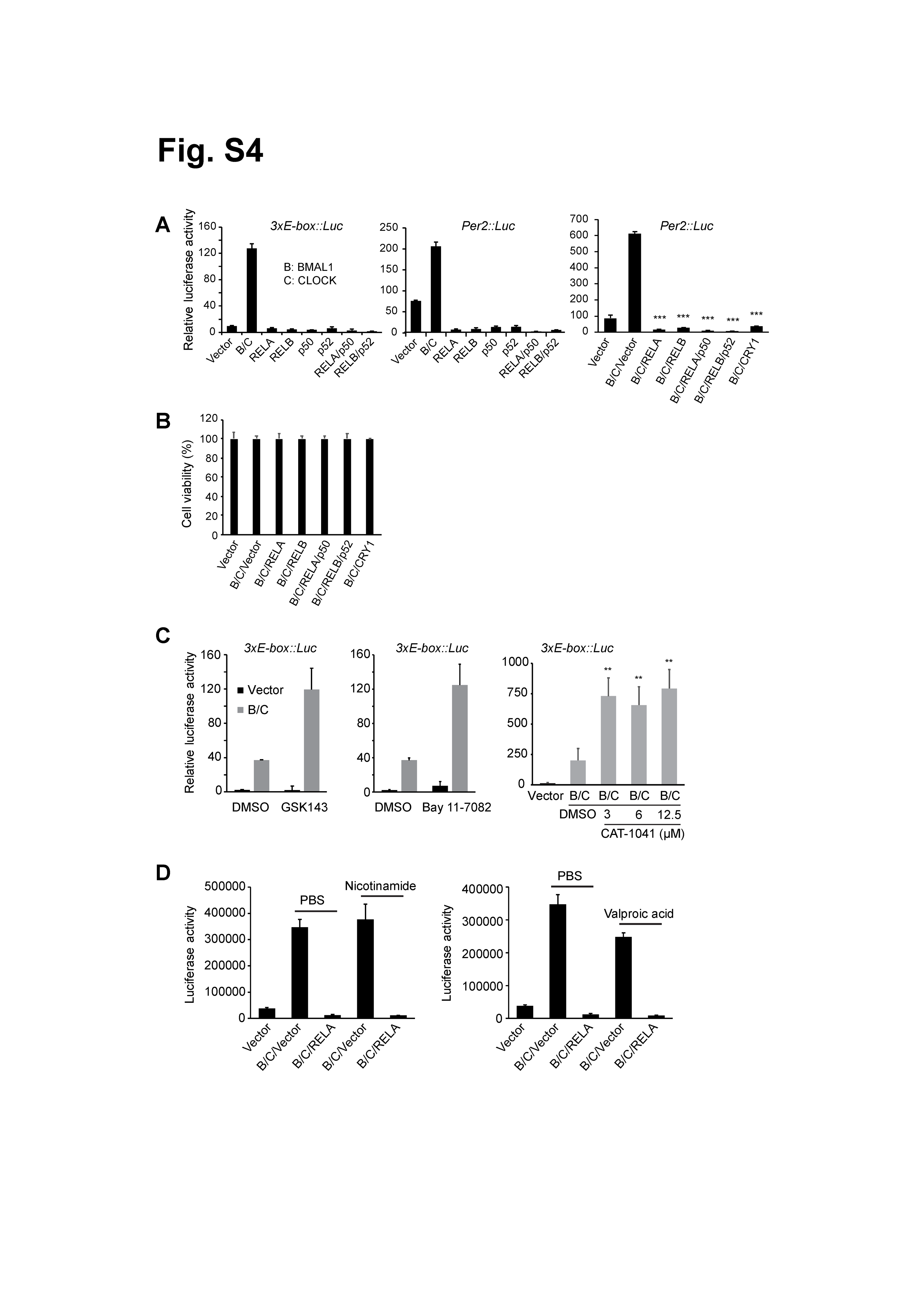

Supplement: S4 Fig — Steady-state luciferase reporter assay in transiently transfected 293T cells. (A) NF-κB itself, in the absence of BMAL1/CLOCK (B/C), did not activate the E-box transcription from the 3xE-box::Luc or Per2-dLuc reporters (left, middle), but repressed BMAL1/CLOCK activation of the Per2::Luc reporter (right). RELA, RELB, p50 and p52 are NF-κB subunits. (B) ATP assay to assess cell viability. Transfection of the NF-kB subunits in 293T cells did not cause toxicity or cell death. Cell samples were collected 24 h post transfection and used for the Dual-Glo Luciferase Assay. (C) Inhibition of the endogenous NF-κB by chemical inhibitors (10 μM GSK143, 20 nM Bay 11–7082, or CAT-1041 as the indicated doses) relieved its repression on the E-box-mediated transcription of the luciferase reporter. (D) HDAC inhibitors nicotinamide (1 mM) and valproic acid (10 mM) did not affect NF-κB repression of E-box transcription. n = 6 independent wells for each assay. ** p < 0.01. (TIF) [file pgen.1009933.s004.tif]

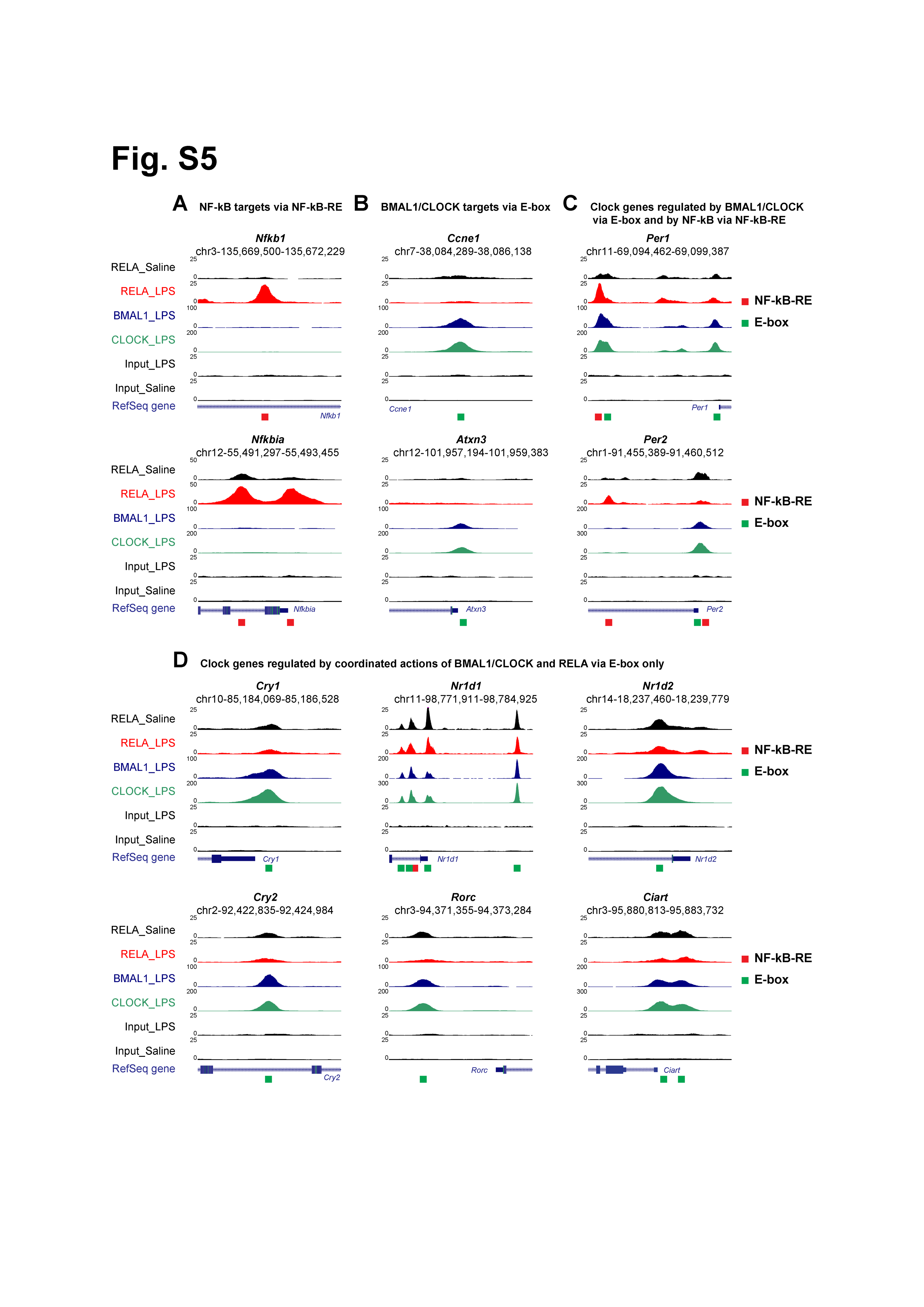

Supplement: S5 Fig — ChIP-seq analysis revealed overlapping binding of BMAL1, CLOCK and RELA at the E-boxes of the indicated clock genes. (A) Examples of NF-kB targets via NF-kB-RE. (B) BMAL1/CLOCK targets via E-box. (C) Examples of core clock genes regulated by BMAL1/CLOCK via E-box and by NF-kB via NF-kB-RE. (D) Examples of core clock genes regulated by coordinated actions of BMAL1/CLOCK and RELA via E-box only. Normalized tag counts are indicated on the Y-axis. The genomic DNA for the region (chromosome # and sequence start and end position) is shown on the top. The predicted motif are shown on the bottom. Green square, E-box. Red square, NF-kB-RE. Motif prediction is in S1 Table. Note that although motif search for Nr1d1 predicted an NF-kB-RE site at the second peak, the sequence is located at the peak periphery, not at the peak center (S1 Table), and the peak was not LPS inducible, suggesting that NF-kB binding at the second peak is mediated by the two E-boxes at the peak center. (TIF) [file pgen.1009933.s005.tif]

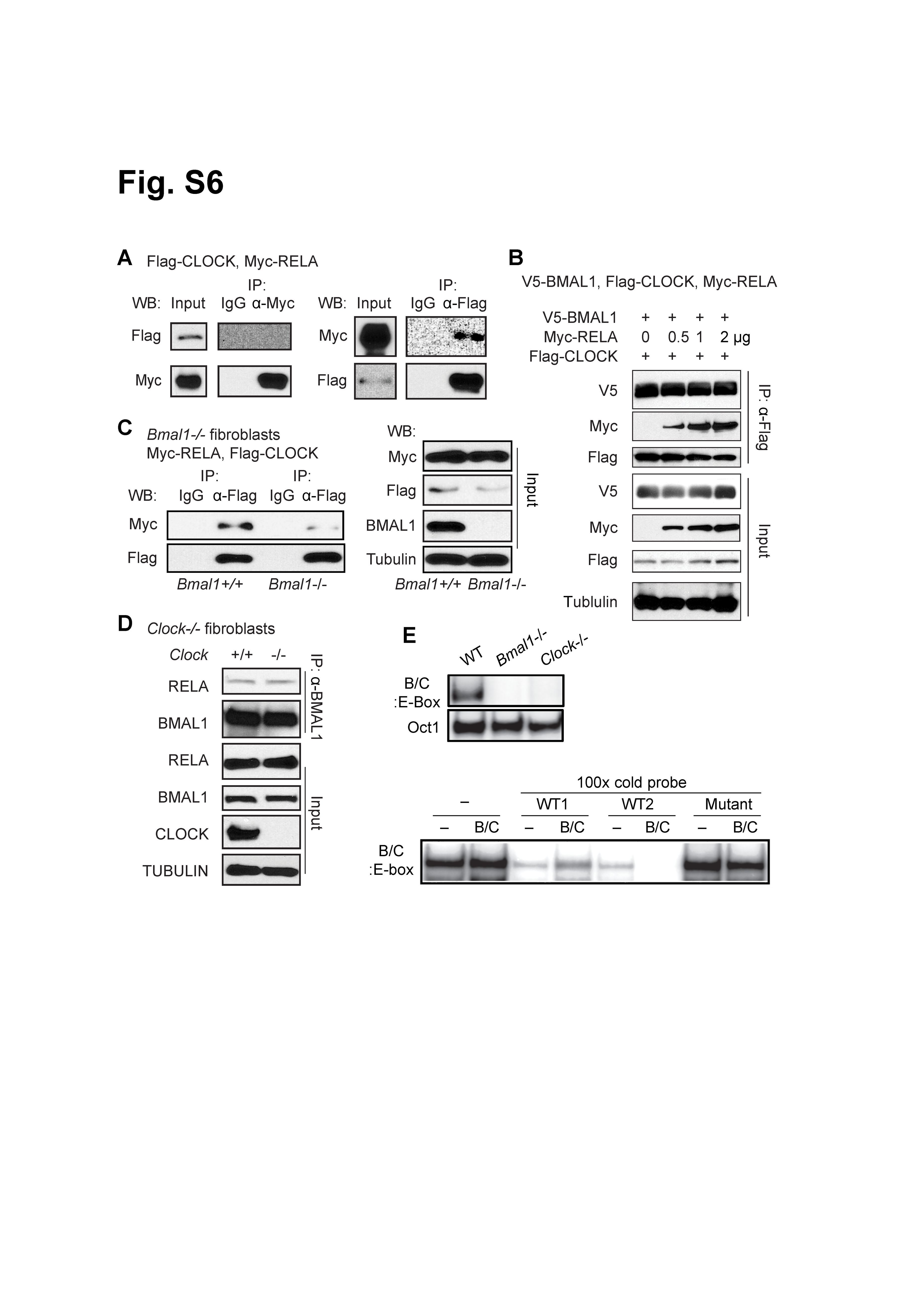

Supplement: S6 Fig — (A) Reciprocal co-IP and Western blot to detect the interaction between RELA and CLOCK. Their interaction was not consistently detected in cotransfected 293T cells even after long exposure, indicative of weak interactions. (B) Interaction between RELA and CLOCK was readily detected in co-transfected 293T cells in the presence of coexpressed BMAL1. (C) The RELA and CLOCK interaction was drastically reduced in Bmal1-deficient mouse fibroblasts. (D) Co-IP and Western blot detected BMAL1 and RELA interaction in Clock-deficient mouse fibroblasts. (E) Electrophoretic mobility shift assay (EMSA). The BMAL1/CLOCK (B/C) heterodimer formed a complex with 32P-labeled E-box duplex probe (B/C:E-box) in WT cells (left), but not in cells deficient in Bmal1 or Clock, indicating that the BMAL1/CLOCK dimer is required for the BMAL1/CLOCK:E-box ternary complex formation. The specificity of complex formation was confirmed by completion with a 100-fold excess of unlabeled (cold) wild type probe (WT1, 2), but not with mutated duplex (mutant). (TIF) [file pgen.1009933.s006.tif]

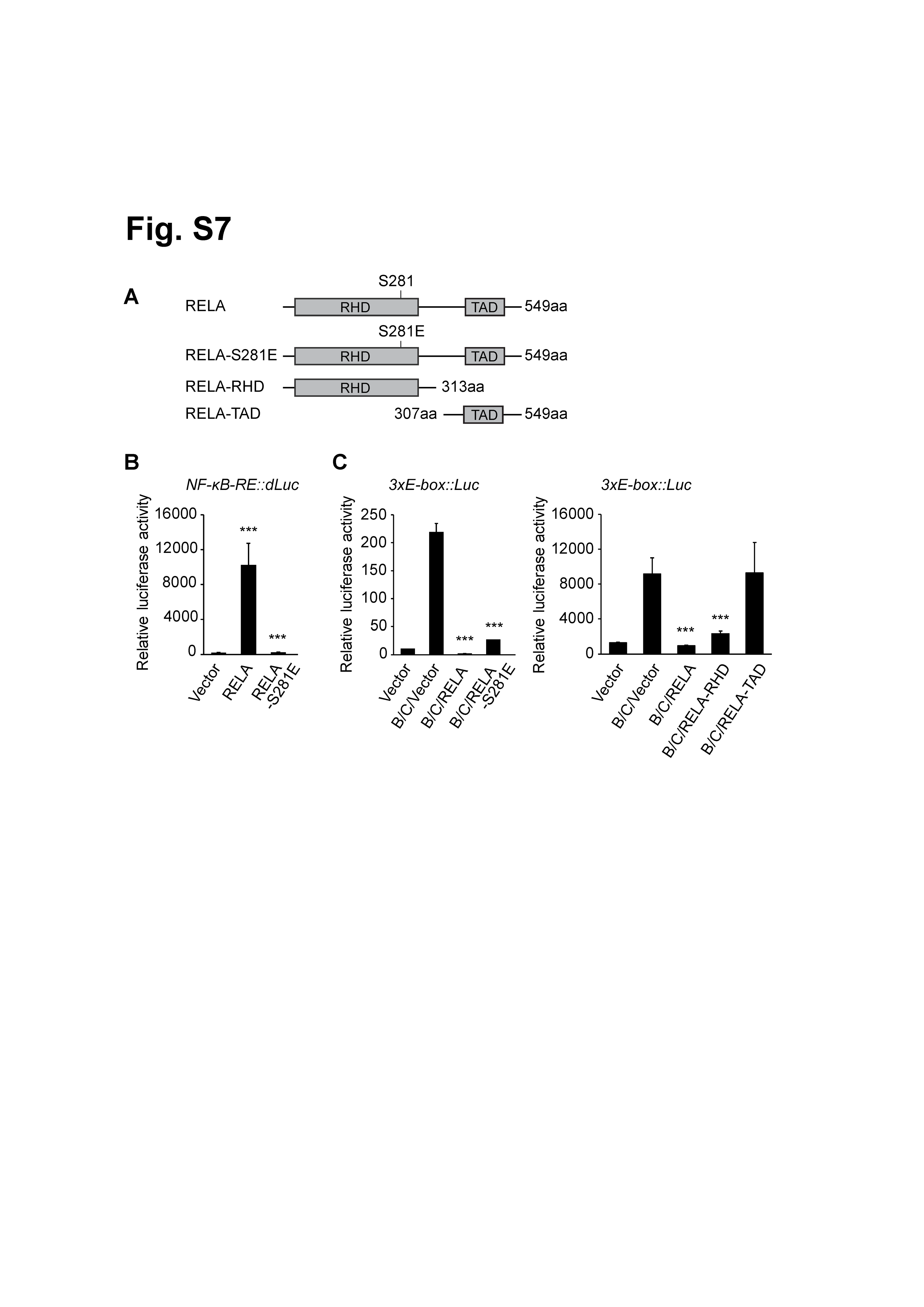

Supplement: S7 Fig — (A) Schematic diagram of domain structure of RELA. RHD: Rel homology domain. TAD: transactivation domain. Shown are the point mutation and truncation constructs used in the study. (B-C) Steady state luciferase reporter assay in transiently transfected 293T cells using the NF-κB-RE::dLuc (B) or 3xE-box::Luc reporter (C). The RELA-S281E mutant failed to activate the NF-κB-RE::dLuc reporter, but effectively repressed the 3xE-box::Luc reporter. The RELA RHD retains the E-box repression activity. n = 6 independent wells. *** p < 0.001. (TIF) [file pgen.1009933.s007.tif]
