# Supplementary material for: NF-κB modifies the mammalian circadian clock through interaction with the core clock protein BMAL1
Source: PLoS Genet. 2021 Nov 22;17(11):e1009933. doi: 10.1371/journal.pgen.1009933 (PMC8648109; doi:10.1371/journal.pgen.1009933)
Supplement: S1 Table — (PDF) [file pgen.1009933.s008.pdf]

S1 Table

The E-box and NF-kB-RE motifs within the binding peaks of BMAL1, CLOCK and RELA in ChIP-seq data

Peak info: PeakID, Chromosome #, Start seq, End seq, Strand, Peak Score, Focus Ratio/Region Size, Annotation, Detailed Annotation, Distance to TSS, Nearest PromoterID, Entrez ID, Nearest Unigene, Nearest Refseq, Nearest Ensembl, Gene Name, Gene Alias, Gene Description, Peak nucleotide sequence. Motif search was done using the JASPAR program with a relative profile score threshold of 85% or above. Red, NF-kB-RE. Green, E-box.

A. NF-kB targets via NF-kB-RE

Nfkb1

Merged-chr3-135670787-1 chr3 135670696 135670878 + 37.400002 p65 intron (NM_008689, intron 1 of 24) NA 20760 NM_008689 18033 Mm.256765 NM_008689 ENSMUSG00000028163 Nfkb1 NF-KB1|NF-kappaB|NF-kappaB1|p105|p50|p50/p105 nuclear factor of kappa light polypeptide gene enhancer in B cells 1, p105

**TTGCAAAAGCAAGTTTATACCTAAAATGTTGAAATATCGACTAAGCTTTGGAGCTGGGGATGAGAAAGCAGTGGGAAAGTCCCTTATGTACCCCTGCTTTCTCAGC
CTTGCCCCCACTTTCCTTTTGTGCTTTTGAGGAAGGACCTTGAGACACAAAGGGAGACACAAGCCTGGGTCTCATT**

Nfkbia

Merged-chr12-55492723-1 chr12 55492632 55492814 + 63.5 p65 promoter-TSS (NM_010907) NA -76 NM_010907 18035 Mm.170515 NM_010907 ENSMUSG00000021025 Nfkbia AI462015|Nfkbi nuclear factor of kappa light polypeptide gene enhancer in B cells inhibitor, alpha

**GGCAGGGGATTTCTCAGGGGCGGGGTCAGGCGCGGGGAATTTCCAAGCCAGTCAGACTAGAAAAAGAACTGGCTCGTCCTCCACTGAGAAGCCTAAACCCAGGGCC
GGGGTTCATCGGAGAAACTCCCTGCGATGAGCCACTGGGGTCATGCACAGGGAACTTTTTGATGAGCCCTGAGTGG**

Merged-chr12-55491939-1 chr12 55491848 55492030 + 72.900002 p65 TTS (NR_035430) NA 708 NM_010907 18035 Mm.170515 NM_010907 ENSMUSG00000021025 Nfkbia AI462015|Nfkbi nuclear factor of kappa light polypeptide gene enhancer in B cells inhibitor, alpha

**GGAGGTGGGGTTGGAGGGGGGATTGCGCAAGGCCGGGGTTTCTGGAGGCCGCGGCCGCGGTGTTTTCCGCGAGGTTATTATGAGCTGAGTGTTCCTGGCAGCCGCC
CAGGGACTTTCCGTGCCCCACCCCCTCCGGGCGGGCGCCGGCCAGACCGGCCCGCCCTCCGCCGCCCGGAACGCCC**

B. BMAL1/CLOCK targets via E-box

Ccne1

Merged-chr7-38085213-2 chr7 38085121 38085306 + 123.450005 Clock|Bmal1 Intergenic CpG 22277 NM_007633 12447 Mm.16110 NM_007633 ENSMUSG00000002068 Ccne1 AW538188|CycE1 cyclin E1

**GCAGCCGCAGAGGACATGGGGCGGGGACAGGGGCGGGGTCGGATCGGAGGCGGAGGCTGGGTGGGCGGGCCCTGGAGTCACGTGTGTAAACACGCCGCACGTGGAG
CTCAGCCTGAGCCAGAAGGGCGGGGCTGCGGGTTCCGAAGGAGGAGGCGGGTAAGGTGCGCGCGCGCGCGCGCATGGAG**

Atxn3

Merged-chr12-101958288-2 chr12 101958179 101958398 + 67.949997 Clock|Bmal1 promoter-TSS (NM_001167914) NA -45 NM_029705 110616 Mm.271914 NM_029705 ENSMUSG00000021189 Atxn3 2210008M02Rik|AI463012|AI647473|ATX3|MJD1|Mjd|Sca3|ataxin-3 ataxin 3

**GCCCCGGCGCGGAGCAGCCCCCTCCAGCAGCTCCGCCCCCGGCCCCACCCCTCAGCCCCGGTCCGCCCCGCCCTAGCCACGTGCCTCTCGCCAACCAATCACTACT
GCGGGCGCACGTCACGAGGCACGCGAACCCGGGAGCACGGGACACGTGACCTGGGCGGCGTTGGTCCTCCCCGCGGCAAGCGTGATGCTGCGGCGCCGCCTGCTGT
GCGGAGG**

C. Clock genes regulated by BMAL1/CLOCK via E-box and by NF-kB via NF-kB-RE

Per1

Merged-chr11-69094874-3 chr11 69094771 69094986 + 115.666664 p65|Clock|Bmal1 Intergenic Intergenic -4078 NM_011065 18626 Mm.7373 NM_011065 ENSMUSG00000020893 Per1 Per|m-rigui|mPer1 period circadian clock 1

**CCTGCGTACAAGTCGCTCGGCACGCCTGCGCAGACTCCGCAGTATTGGGTAAGTGTCGTCAAGGAAAATCCCCAGCTTCTGGGTAAACAAGTTGCCGCGTGAGCCA
GCCTGCACGTGTTCCCTACAGCTCTGAGCCCTCTCAGCCTATGAGAAAGTTTTTAGGGCAGGGCTGGCATTTGCGTCACTGATTTAGGCAGGGCGGGGTTGTCTCT
GCA**

Merged-chr11-69098809-2 chr11 69098703 69098915 + 114.699997 Clock|Bmal1 promoter-TSS (NM_011065) NA -147 NM_011065 18626 Mm.7373 NM_011065 ENSMUSG00000020893 Per1 Per|m-rigui|mPer1 period circadian clock 1

GACGGTGTGAGACATCCTGATCGCATTGGCTGACTGAGCGGTGTCTGAGGCCCTTCAGCCCAGCACCAGCACCCAAGTCCACGTGCAGGGATGTGTGTGACACAGC
CCTGACCTCAGTGGGGGCCAGTAGCCAATCAGATGCCAGGAAGAGATCCTTAGCCAACCGGGGGCGGGGCCTGCGGCTCTTCGGGCAGAAGGCCAATGAGGGGCAG

Per2

Merged-chr1-91459395-3 chr1 91459274 91459506 + 122.003334 p65|Clock|Bmal1 promoter-TSS (NM_011066) promoter-TSS (NM_011066) -62 NM_011066 18627 Mm.482463 NM_011066 ENSMUSG00000028976 Per2 mKIAA0347|mPer2 period circadian clock 2

CGCTGCCGCCGCGTCGCCCTCCGCTGTCACATAGTGGAAAACGTGACCGCGCGCGCTGAGCCCGCCCCTGCGCGCGCCATTGGTCGGAGTGCCACCTCATTTGCAT
ACTGGCGGGGCGGAACCTGAGAGCTACGCTCATCAATTGGTGGAGACGCCCGTCGCTCTCTTTACATAAGACGCACATGGAACTCCATGTTCGCCGCCCGGGTTCC
TCAATGAAGATGCTGCGCGC

Merged-chr1-91456120-1 chr1 9145602991456211+ 18.700001 p65 intron (NM_011066, intron 1 of 22) NA 3208 NM_011066 18627 Mm.482463 NM_011066 ENSMUSG00000055866 Per2 mKIAA0347|mPer2 period circadian clock 2

TCGCTTTCCTCAGCCAGACAGAGGAAAAGAGCCCTGGGTGATGAGATCATGGGTCTGGGGTGGGGGAGGGTACCCCAGGCTGGCTGCTGGGAGTTCACAGTATTGC
TCTCTCAGCCTCTGCAGCTAGCTACACTTTCCACAAGAAATGGTTAAAAACACAGCAAGCCACGATGACAAAACAG

D. Clock genes regulated by coordinated actions of BMAL1/CLOCK and NF-kB via E-box only

Dbp

Merged-chr7-45704969-3 chr7 4570485045705083+ 152.96666 p65|Clock|Bmal1 promoter-TSS (NM_016974) promoter-TSS (NM_016974) -281 NM_016974 13170 Mm.24222 NM_016974 ENSMUSG00000059824 Dbp - D site albumin promoter binding protein

AAGTGGGTCACGGTCCCGCCTCCAGCGCCTCCTCCCATTGGCCAAATATAGGTCAGTGTCCCGCCTTCTTCCAAAAGGCGAGGCAACTACACAAGTTCAGCCCCTC
ACGCGCCTCCAGGGTCTTGGCATATCCCTTCATCTCATTGGCTGAGCGTCTCAAGGGCTCGAGACGTGATTGGTTCTTGCCAGAAGCCGGAGGAAGGCACGCGGCT
GGGGTGCATGGCCCAAGTGGA

Merged-chr7-45706063-2 chr7 4570596545706161+ 139.449997 Clock|Bmal1 intron (NM_016974, intron 1 of 3) NA 816 NM_016974 13170 Mm.24222 NM_016974 ENSMUSG00000059824 Dbp - D site albumin promoter binding protein

GCCAGACCCCTCTTTCTTTCCTTTGGCCTCAGAGTCTGGACCCCTGGAGTCCTAGGGGAAGGGAGGGGGTGTGTCGGATGCTCACACGGTGCAGACACGGGCTTGG
GCGGGGCCTCGCAGGGTCACATGGAAACTAGGGATGAGGAATGTGCCTGAGCAGTGGAGCCCCTTCCAGCCCCAACAGACTGTGGGGGAG

Merged-chr7-45707753-3 chr7 4570765345707852+ 272.633331 p65|Clock|Bmal1 intron (NM_016974, intron 2 of 3) intron (NM_016974, intron 2 of 3) 2505 NM_016974 13170 Mm.24222 NM_016974 ENSMUSG00000059824 Dbp - D site albumin promoter binding protein

GGTACACCTGTCCCTGATCCGACCTCCAGGCAAAAACCAGTGCTGCACATTCCCTCGCCACGTGAGTCCGCCTTCCTAGCTGCGCGCACGCGCTCTCACGCGCGAG
GAGGCGGGGCCGGGAACGTGCGGGTGCTTGGTTGGGGAGGAGGGGAAAGGGGAAGCTGTAGGCCACACGTGATGCGGCCCTGAACTGGTGTCC

Per3

Merged-chr4-151044770-3 chr4 151044631 151044898 + 99.816666 p65|Clock|Bmal1 promoter-TSS (NM_011067) promoter-TSS (NM_011067) -99 NM_011067 18628 Mm.121361 NM_011067 ENSMUSG00000028957 Per3 2810049O06Rik|mPer3 period circadian clock 3

CAGAGCCTCGCCCACCCGCGGGCAGTCCAGTCTCGAGGCCGTCCGCGTGCCCAGTGGCCGCCGGCCCCGCCCCCTGCGCGCCAGGGATCCGGTCCCGCCCCTTCGC
CGCGCGATTGGTCGCTGTTACCGGCAACACGAGCTCCCATTGGTCAGAGGGCGGGGTGCAGGTCGTGCACGGCGGGGGTTACATAACCGCGGGCGGGTGGGCGGGT
GCAAAGGCCCCCGAGTACCTTACATAACGCGCGGGTCGGGCGAGGGCGGCTCTCG

Cry1

Merged-chr10-85185299-3 chr10 8518498085185421+ 103.053329 p65|Clock|Bmal1 promoter-TSS (NM_007771) NA -244 NM_007771 12952 Mm.26237 NM_007771 ENSMUSG00000020038 Cry1 AU020726|AU021000|Phll1 cryptochrome 1 (photolyase-like)

AGCGCGGGCTAGAGCTCCAGGCCGCGGGACGCTCGCCTGCCGGTCACCGGTCCCGAGGCTGCCCGGGTGACTCCGCCGCGTCAGCGGCCTCCGCCCCGCCCCCGGC
TTCTCATTGGGCGGCATGCACCCCACGTGACCACCGGCACCTCACGTTTCTGAAGTGTGTTTACTACACTGGCTCAGACCCCCGCGTGCGCCGCCGCGCCCGGTGC
TTCGGGACCGGTTGCGATCGCTGGGCGCTCACGACCTGAGTTCTCGCGTGGGATCCGGACTCGCAGCCTCTGCGGGTCTCCTGAGCCCCAGGCCGCTCGCGGGGGC
ACAGGCCGCGAGAAGGGGGAGGAGGCATAGTTCTGCTCCCGGGGCCGGGAGCCGCCTCCATTGGCCGAGGCTCGCGGGGACCCGGATGAGCACGGGGCTGCGGGGA
GCTCGCGGGCGCGCGCT

Cry2

Merged-chr2-92423909-2 chr2 92423802 92424017 + 131.100006 Clock|Bmal1 intron (NM_009963, intron 3 of 11) NA 10160 NM_009963 12953 Mm.254181 NM_009963 ENSMUSG00000068742 Cry2 AV006279|D130054K12Rik cryptochrome 2 (photolyase-like)

GTGCTGCCCCCATTAGCCCAGATAGGAGCTGGCTGCTGCCTCTGCAGTCCCTGCCCTCCCCTGGCACGTGGCAGGGGAAGGGCTCCACAGCAAGCCTCTCCTCCTG
AGGGCTTGGCTCACGTGCACAGCTGTGTGACCCAGCCTCCTGGCAGCCAGCCCTGGGTTGGGAGGAGGGCAGGGAGCAGTAGCAGCTACAGAGGGTGGCATCTCTA
GTA

Nr1d1

Merged-chr11-98773066-2 chr11 98772972 98773160 + 134.300003 Clock|Bmal1 intron (NM_145434, intron 1 of 7) NA 2311 NM_145434 217166 Mm.390397 NM_145434 ENSMUSG00000020889 Nr1d1 A530070C09Rik|R75201 nuclear receptor subfamily 1, group D, member 1

CACCTCTGCCAAGTACTTAGCCTGGCCGGCCATGGGTGGAGTGGGGAAAGGGGAGGATCCTCAGACACGTGTGTGAGGTGACACATGCCATGCCACCGAGTCGACA
GAAGCACATGTCTTGCTCACCCACTGACACACACTAAACCAGGCAGGGCAACCTTAAAATAGCTCAAGGTTGGTCAGGGTGA

Merged-chr11-98773862-3 chr11 9877374698773969+ 177.233337 p65|Clock|Bmal1 intron (NM_145434, intron 1 of 7) CpG 1520 NM_145434 217166 Mm.390397 NM_145434 ENSMUSG00000020889 Nr1d1 A530070C09Rik|R75201 nuclear receptor subfamily 1, group D, member 1

TGAGAGGAAAAGAAGCCAGGCGGGGGCGGTGAGTCAGCCCAGCTTCACCCAGATCTAGGCGGAGAAGGGCGGGGCGGGAGCCTGGCCCTCAGCTGGCCTCACGTCC
CCACTCCACGTGTGCCCCTCGCACGTGGCACCCCAACGAGGAACCCCGGAACTCGAAGCACCGCCTTCTCCGCTTCCCTTCTGTCGCAAGGAAAACCCCGCCCTCG
CCCCGCCCCTC

Merged-chr11-98775381-3 chr11 9877526298775525+ 113.599998 p65|Clock|Bmal1 promoter-TSS (NM_145434) promoter-TSS (NM_145434) -16 NM_145434 217166 Mm.390397 NM_145434 ENSMUSG00000020889 Nr1d1 A530070C09Rik|R75201 nuclear receptor subfamily 1, group D, member 1

AGCCTTTCCCTGGGACAGAGGGCTCTGCGCAGGCGCCAGCAGCCCAGGGTTCCCGGGCCGCACGCGGCACTGGAGCAGGTACCATGTGATTCCAGGGAGCGCCTCG
TGCCCCAGTGACACACTTTTCCAGCAGCCGGCACGTTGAGATCTGTGCGCCTGCGCACCGCCAGGATTGTTGGGATTTGTAGTCTACTGACAATGTAGGTGGAGTG
TGCCTATTCCTTTTCTGCTTTGCAAAAGTGCTTGCTGGCAGCGGGGAGGAG

Nr1d1 peak
Merged-chr11-98783508-3 chr11 9878339698783611+ 280.100006 p65|Clock|Bmal1 Intergenic Intergenic -8126 NM_145434 217166 Mm.390397 NM_145434 ENSMUSG00000020889 Nr1d1 A530070C09Rik|R75201 nuclear receptor subfamily 1, group D, member 1

GCTGTCTTGAAGCTCACGCGGGCTGCCCGGGTCACATGGCACGGGCATGTGCAGCCTGCTCCATTTCTACCACAACAACTCGCTCATAAACAGCCCGGCTGCTGCG
GCGGTGGCGGCGGAGACATGTGCAAGTGAGGGGGTGGGGCACGTGGCCCTGCCCGAGCCAAGGGAAGGACTGGATTGCTCAGCGCTGCCTCCACCCCTGCCCGCAC
TGG

Nr1d2

Merged-chr14-18238620-3 chr14 18238503 18238735 + 219.96666 p65|Clock|Bmal1 intron (NM_011584, intron 1 of 7) CpG 487 NM_011584 353187 Mm.26587 NM_011584 ENSMUSG00000021775 Nr1d2 RVR|Rev-erb nuclear receptor subfamily 1, group D, member 2

CTCCCCCCGCGGCACCGCCCCCACGTGTTCCTCCGGCCCCGGGTAATGTGGGTCGCCCCGCGGCCTCACGTGTCTCCGCCCGCCACCACCCAGCTCGCATTATGTA
ATGCTGCGTCACTCGCCCGCGCAGCCAACCAGCGCCCGCGCCGCCGAACGTAAACACACCGTGCACGTGGCCAGTGCGGCAGGGCCATGTGAGGGGGGCGTCCGGC
GCGCGGGGAGCCCGAGTGCG

Rorc

Merged-chr3-94371815-2 chr3 9437171994371912+ 95.149994 Clock|Bmal1 promoter-TSS (NM_011281) NA -979 NM_011281 19885 Mm.4372 NM_011281 ENSMUSG00000028150 Rorc Nr1f3|RORgamma|TOR|Thor RAR-related orphan receptor gamma

TGGAGAATGGAATGCTGGGAGCGAAGGGACAGCTGCCTGCCCTCTCCACGCCAGGTGCCACGTGCACCAGGTGGAGGGAGTGGGCGAGTCACGAGGCCCTGGCGTG
GCCGGCTCCTGCCCTGCTGTTTACCAGCTGGAGAAAGCAGGAGGAGGGGTTGGGAGCCTCCTGCTCCCAGCCTGGCCCCTAGCCAGC

Ciart/Chrono

Merged-chr3-95882288-3 chr3 9588212695882906+ 121.550003 p65|Clock|Bmal1 promoter-TSS (NM_001033302) promoter-TSS (NM_001033302) -44 NM_001033302 229599 Mm.331218 NM_001033302 ENSMUSG00000038550 Ciart Chrono|Gm129 circadian associated repressor of transcription

GGAGAGCGCTTGGCTCTCGTCCGCTTACCTGGGTCTGTCGGTTCAGCCCCGGTCGGTATGCAGCTTCTTCACTGTCTGTGCTGGTCTAGCCGACCCACCTCCGCCT
CACTCTGTGGGTCCCGCCCCTTCCCCCTCCCAGACACGTGCGGTTCTGCACGTGCCTCCCCCTGCGTACACAGACCTTGTGCTCTCATTGGATAGCTTACCGGCAA
CACGTGACTCTAGATGCAGACGCTCACAGCCCATTTGCTACGGCACGCGGGGGCTCGGCCAATAGAGAGGCAGCCAAGTGGTGATTGGCTGGAAGGGAAGCAGTCC
ATGCTCCGCCCGTACATGCGGCTCCTCGGGTGATAAAAAAGGAGGATGGAAAAAAGTGAAAATTAAGGAAGGGGCGGAAAAGGGTGGAGCTCATAGCCTTAAATAG
GACAGCACTGCGCGGGAGACGCACGGCTGGAGTGTACAGAGCGGGGCCGAGCCACGGAGGTGGCGCCTCGGGACGTGGGCCCCTCTGAGCGCCTCCGCGCACGGGC
GCAGCACGTGGGAGCCCTCAGCAACTCGTGTCCGCAACGTGTGTGACAACGGACGTGTGACGCCGGGCATCCGTTCCCTTGTCGGCGGCTCGCCCTCGTGACTCCC
CACTCTTTCATCCTCCCCTCCTCGCCACCAAGTTGGGTGCCTCCTGCCCTGCTGGGCGAGGAGGGTGGGGACTGCGGCTGGAACACCGCGTCCTCTCCGGGGCTCC
CGCCGCGCAGCTTCTCCAGCCGGTTCTTACATAACTTGC
