# Supplementary material for: NF-κB modifies the mammalian circadian clock through interaction with the core clock protein BMAL1
Source: PLoS Genet. 2021 Nov 22;17(11):e1009933. doi: 10.1371/journal.pgen.1009933 (PMC8648109; doi:10.1371/journal.pgen.1009933)
Supplement: S2 Table — (PDF) [file pgen.1009933.s009.pdf]

S2 Table. Q-PCR primers for human genes

PER3-F	TTTCCTAATGTCGCCGAAGAG
PER3-R	CCTGGTATGTCATGAGAATGCG
RoRα1-F	GAGGTATCTCAGTCACGAAG
RoRα1-R	AACAGTTCTTCTGACGAGGACAGG
GAPDH-F	TGCACCACCAACTGCTTAGC
GAPDH-R	ACAGTCTTCTGGGTGGCAGTG
BMAL1-F	GCCCATTGAACATCACGAGTAC
BMAL1-R	CCTGAGCCTGGCCTGATAGTAG
CLOCK-F	GGCACCACCCATAATAGGGTA
CLOCK-R	TGTTGCCCCTTAGTCAGGAAC
PER1-F	TCTGTAAGGATGTGCATCTGGT
PER1-R	CAGGCAGTTGATCTGCTGGT
PER2-F	AGTTGGCCTGCAAGAACCAG
PER2-R	ACTCGCATTTCCTCTTCAGGG
CRY1-F	ACAGGTGGCGATTTTTGCTTC
CRY1-R	TCCAAAGGGCTCAGAATCATACT
CRY2-F	CGTGTTCCCAAGGCTGTTCA
CRY2-R	CTCCGTCACTACTTCCACACC
DBP-F	GTTGATGACCTTTGAACCCGA
DBP-R	CCTCCGGCACCTGGATTTTT
NR1D1-F	TGGACTCCAACAACAACACAG
NR1D1-R	GTGGGAAGTAGGTGGGACAG
NR1D2-F	CAGCAATGTCGCTTCAAAAA
NR1D2-R	TGGTCTTCATTGCACTTTGC
RORC-F	TGAGAAGGACAGGGAGCCAA
RORC-R	CCACAGATTTTGCAAGGGATCA
IL6-F	ACTCACCTCTTCAGAACGAATTG
IL6-R	CCATCTTTGGAAGGTTCAGGTTG
TNFα-F	CCTCTCTCTAATCAGCCCTCTG
TNFα-R	GAGGACCTGGGAGTAGATGAG
