# Supplementary material for: NF-κB modifies the mammalian circadian clock through interaction with the core clock protein BMAL1
Source: PLoS Genet. 2021 Nov 22;17(11):e1009933. doi: 10.1371/journal.pgen.1009933 (PMC8648109; doi:10.1371/journal.pgen.1009933)
Supplement: S3 Table — (PDF) [file pgen.1009933.s010.pdf]

S3 Table. Q-PCR primers for ChIP-PCR

Per2 E-box-F	GGAGTCGCGGCCAATG
Per2 E-box-R	CCACAGCTGCACGTATC
DBP E-box-F	CCTGGGCACACCTGCTC
DBP E-box-R	AGGAGGGATGGGAGGACTCA
