# Supplementary material for: NF-κB modifies the mammalian circadian clock through interaction with the core clock protein BMAL1
Source: PLoS Genet. 2021 Nov 22;17(11):e1009933. doi: 10.1371/journal.pgen.1009933 (PMC8648109; doi:10.1371/journal.pgen.1009933)
Supplement: S4 Table — (PDF) [file pgen.1009933.s011.pdf]

S4 Table. Probe sequences for gel shift

WT1 probe E-box-top	CGCGCAAAGCCATGTGCTTCCCCCT
WT1 probe E-box-bottom	AGGGGGAAGCACATGGCTTTGCGCG
WT2 probe-E-box-top	GGGACACGTGACCC
WT2 probe-E-box-bottom	GGGTCACGTGTCCC
Mutant probe E-box-mutant-top	GGGACAGCTGACCC
Mutant probe E-box-mutant-bottom	GGGTCAGCTGTCCC
